# Inclusion flotation-driven channel segregation in solidifying steels

**DOI:** 10.1038/ncomms6572

**Published:** 2014-11-25

**Authors:** Dianzhong Li, Xing-Qiu Chen, Paixian Fu, Xiaoping Ma, Hongwei Liu, Yun Chen, Yanfei Cao, Yikun Luan, Yiyi Li

**Affiliations:** 1 Shenyang National Laboratory for Materials Science, Institute of Metal Research, Chinese Academy of Sciences, Shenyang 110016, China

## Abstract

Channel segregation, which is featured by the strip-like shape with compositional variation in cast materials due to density contrast-induced flow during solidification, frequently causes the severe destruction of homogeneity and some fatal damage. An investigation of its mechanism sheds light on the understanding and control of the channel segregation formation in solidifying metals, such as steels. Until now, it still remains controversial what composes the density contrasts and, to what extent, how it affects channel segregation. Here we discover a new force of inclusion flotation that drives the occurrence of channel segregation. It originates from oxide-based inclusions (Al_2_O_3_/MnS) and their sufficient volume fraction-driven flotation becomes stronger than the traditionally recognized inter-dendritic thermosolutal buoyancy, inducing the destabilization of the mushy zone and dominating the formation of channels. This study uncovers the mystery of oxygen in steels, extends the classical macro-segregation theory and highlights a significant technological breakthrough to control macrosegregation.

## Abstract (web summary)

A long-term problem in the casting of alloys such as steels is macrosegregation, which are variations in composition of the cast material. Here, the authors observe that the formation and flotation of oxide-based inclusions from light metals represent a mechanism through which macrosegregation occurs in steels.

## Graphical abstract

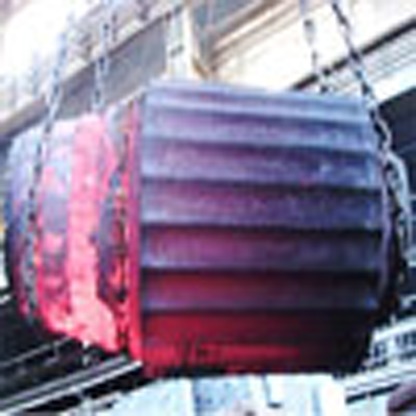

Defects in materials often cause various failures in the service process. A typical defect, which is referred to as macrosegregation^1^,^2^,^3^,^4^,^5^,^6^, frequently occurs during solidification. It reflects a phenomenon of chemical compositional variation, which appears in the range from several millimetres to centimetres or even up to metres in castings and ingots. Although this defect was initially described in bronze gun barrels nearly half a millennium ago^7^, the theory of macrosegregation^4^,^6^ was pioneered by Flemings and co-workers about half a century ago. Until then, macrosegregation was considered to be a result of gravity-driven flow due to density contrast during solidification. Currently, macrosegregation is induced by three known forces: the buoyancy force of the natural thermosolutal convection^8^, the solid movement force of the grain settling or flotation, and the shrinkage force of the volume contraction during solidification. Macrosegregation is generally classified as various types^4^,^5^,^6^,^9^ based on shape and position. The most typical and severe one is the channel segregation (CS, also referred to as ‘A segregates^4^,^5^,^9^’, ‘freckle^6^,^10^’, ‘chimneys^11^’ or ‘compositional stratification^3^’), which have long been a subject of investigation in the fields of metallurgy, geophysics^12^,^13^ and geology^14^. CS in steels was substantially attributed to the inter-dendritic thermosolutal buoyancy force due to the density contrast between solute-enriched melt and bulk liquid^15^,^16^, according to the classical macrosegregation theory^4^,^6^. In this context, the corresponding modelling^5^,^10^,^17^,^18^,^19^,^20^,^21^ was developed to simulate the formation of macrosegregation.

Given the fact that classical macrosegregation theories have been extensively accepted and well-documented in the textbooks, there seems no reason to suspect their incompleteness. However, it does exist^6^. Based on these theories, CS will unavoidably appear in large steel ingots as the inter-dendritic thermosolutal convection will be very intense due to tall and sufficient sections. For instance, CS definitely occurs in a 100-ton ingot due to an extensive period of solidification (over 25 h) and a slow average cooling rate (approximately ≪5 °C h^−1^; see Supplementary Figs 1,2 and Supplementary Note 1). Unexpectedly, the examinations of our synthesized 100-ton steel ingots with a total oxygen (T.O) concentration of ~1.0 × 10^−3^ wt.% revealed that the CS was not observed in the fully dissected cross-section (as discussed below). This fact does not correspond to the expected occurrence of CS from the classical theories, which motivates the exploration of its mechanism in solidifying steels. Via systematic investigations of various dissected ingots (0.5, 5, 5.8, 14, 16, 20, 69, 100, 234, 535 and 650 (ref. 22) tons; see Supplementary Figs 3–9, Supplementary Table 1, and Supplementary Notes 2 and 3), we have discovered the fourth force of light oxide-based inclusion flotation to drive the formation of macrosegregation. This force is capable of destabilizing the mushy zone by altering the flow fields of their surrounding melts and dominates CS formation. It demonstrates that CS can be significantly reduced or even completely eliminated in subsequent solidified ingots through the upstream low-oxygen purifying metallurgy, which is intrinsically different from traditional methods. From the viewpoint of engineering applications, this study may rapidly yield practical benefits to the annual global manufacture of over 50 million tons of ingots, heavy plates and castings^23^.

## Results

### Experimental characterizations of inclusions and CS

**Figure 1:**
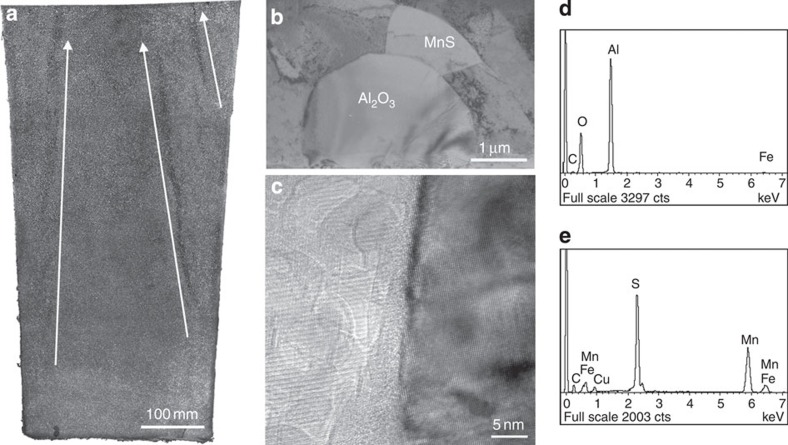
Occurrences of CS and OIs. (**a**) **Experiment I**: The sectioned surface of the Al-deoxidation 0.5-ton ingot has been cut along the axle plane. The surface has been etched by dilute nitric acid to display the microstructures and CS as indicated by arrows. (**b**) The TEM image shows that the MnS combines with the Al_2_O_3_ to form OIs in the region of the CS. (**c**) High-resolution TEM image to analyse the interface between MnS and Al_2_O_3_. (**d**,**e**) The EDS results for Al_2_O_3_ and MnS in **b**.

**Figure 2:**
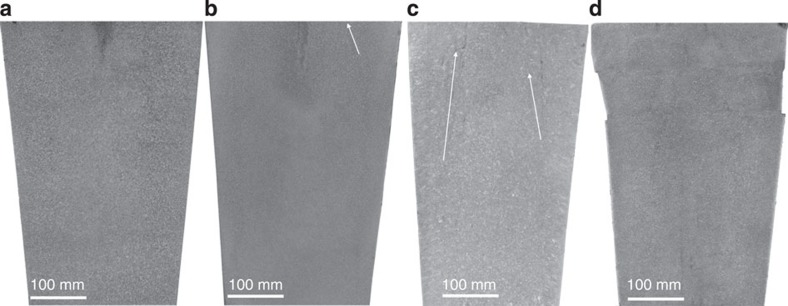
Existence and elimination of CS. The sectioned surfaces of four 0.5-ton ingots have been treated by the VCD technique and cut along the axle plane. (**a**) **Experiment II**: CS disappears with T.O=1.0 × 10^−3^ wt.%. (**b**) **Experiment III**: CS has been significantly reduced with T.O=1.5 × 10^−3^ wt.%. (**c**) **Experiment IV**: CS occurred with T.O=2.0 × 10^−3^ wt.% in the presence of a small amount of OI. (**d**) **Experiment V**: CS has disappeared in this ingot poured in the air by argon protection as long as the oxygen concentration has been limited to a low level T.O=0.7 × 10^−3^ wt.%. The arrows denote the presence of CS.

To address this mechanism, we have designed five Experiments (**I**–**V**), as compiled in Table 1. Substantial differences are observed in the fully dissected, etched longitudinal sections of ingots **I** and **II** (Figs 1a and 2a). Experiment **I** exhibits typical CS with some narrow, vertical and centre-inclined axial-symmetrical strip-like chains (Fig. 1a), whereas the CS disappears in Experiment **II** (Fig. 2a). Their differences are observed in the employed deoxidation techniques and the pouring methods, which result in a highly distinct oxygen concentration. In the former **I,** the T.O is 5.6 × 10^−3^ wt.% with the AD and air pouring treatments, whereas the VCD and vacuum pouring treatments yield T.O=1.0 × 10^−3^ wt.% in the latter **II** (Table 1). The analysis reveals that the inclusions enriched in the CS region of the Experiment **I** (Fig. 1b) are primarily composed of Al_2_O_3_, MnS and minor amounts of bubble-like cavities (see Supplementary Figs 10–16 and Supplementary Note 4). A special feature has been regularly observed in the CS region, in which the majority of MnS (Fig. 1d,e) tends to combine with Al_2_O_3_ (Fig. 1b) to form the Al_2_O_3_/MnS oxide-based inclusions (OIs); they almost have a diameter in the range of ~5~50 μm. The typical morphologies include the MnS-like impurity precipitates surrounding the centred Al_2_O_3_ (Fig. 1c). Some main elements are also promoted to segregate, which corresponds to the occurrence of OIs (see Supplementary Table 2). Compared with Experiment **I**, the amount and size of the OIs, which are dependent on the oxygen concentration, have been significantly reduced in Experiment **II** (Fig. 2a). Thus, the contrasting experiments between **I** and **II** imply that the oxygen concentration seems to be the crucial factor for CS formation.

To elaborate the effects of oxygen in Experiment **III**, we have retained T.O=1.5 × 10^−3^ wt.% with the VCD technique but have adjusted the S concentration to a higher level of 1.3 × 10^−2^ wt.% (Table 1). Similar to Experiment **II**, the amount of CS has been significantly reduced (Fig. 2b), despite the existence of a relatively large amount of dispersed MnS in Experiment **III**. Using the VCD technique, the CS appears as long as a higher concentration of oxygen exists, as elucidated by our Experiment **IV** (Fig. 2c) of a 0.5-ton vacuum poured ingot with a T.O of 2.0 × 10^−3^ wt.%. In contrast, the CS disappears in Experiment **V** (Fig. 2d) with a lower oxygen concentration of the T.O=0.7 × 10^−3^ wt.% (Table 1) by the VCD technique poured in the air by the argon protection. We extended our experiments to a series of significantly heavier ingots (that is, 5, 5.8, 14, 16, 20 and 69 tons; see Supplementary Table 1 and Supplementary Methods). In this similar situation the CS becomes very slight or disappeared only if the oxygen concentration is controlled to a low level (typically, the T.O is ~1.0 × 10^−3^ wt.%), regardless of which methods (AD or VCD, air or vacuum pouring) have been employed. These results clarify that the oxygen concentration has a primary effect on CS formation.

### Multiscale modelling

**Figure 3:**
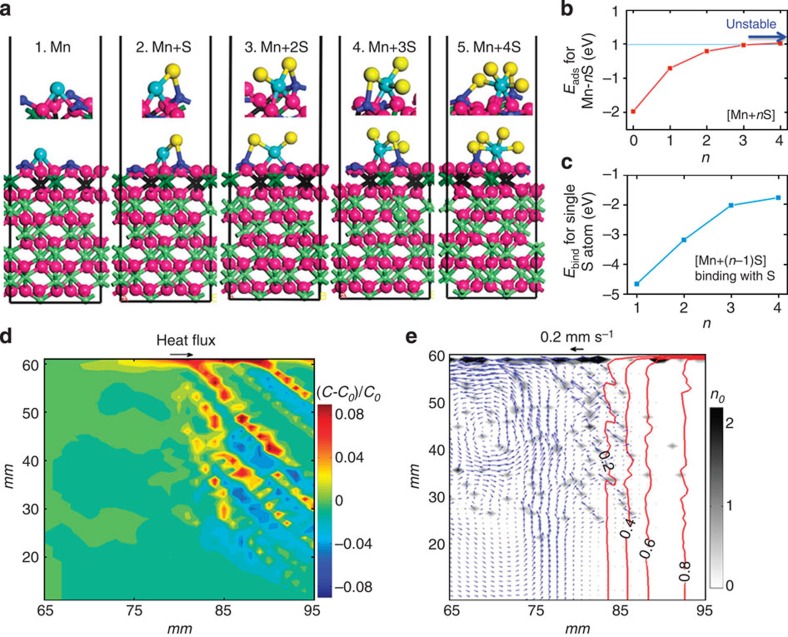
Nucleation of OIs and their inclusion-driven flotation. (**a**) The first principles calculations simulated the nucleating process Mn+*n*S clusters by trapping S and Mn ions on the Al_2_O_3_ (0001) surface. The upper panels denote the local geometric structural details for the Mn or Mn+*n*S adsorptions. Blue and yellow balls denote the Mn ions and S ions, respectively. Depending on the S introduction to the Mn+*n*S atom complex, the bonding length between Mn and the nearest neighbouring O1 atoms has been slightly reduced from *n*=0 to *n*=2 and is subsequently increased for the cases of *n*=3 and 4. This feature corresponds to the stable adsorption for *n*=0, 1 and 2 due to the enhanced attraction between the trapped Mn atoms and the trapped O1 atoms on the surface. The increasing distance for the *n*=3 and *n*=4 cases reveals the weakening of the adsorption. (**b**) The adsorption energies of the Mn+*n*S atom complex on the surface as a function of the trapped S ions. (**c**) The binding energies have been obtained from the Mn+*n*S complex with respect to the extra-S atom and the already formed Mn+(*n*−1)S complex on the Al_2_O_3_ (0001) surface. (**d**) The simulated CS of Fe-0.36 wt.% C steel coupled with the flotation of the initial 500 alumina particles (OIs) with a diameter of 15 μm is unidirectionally solidified in a cavity (100 × 60 mm^2^). (**e**) Detailed information of the liquid flow patterns in the mushy zone and the distribution of solid particles, which demonstrates that the floating particles perturb the flow field by accelerating the local flow velocity and altering its direction. The isolines of the solid fraction are also superimposed in the figure. The legend on the right shows the number of particles *n*_*0*_.

As the dissolved oxygen concentration is ≪1.0 × 10^−3^ wt.% in steel melt treated by the AD technique, the Al_2_O_3_ in the CS would most likely not form via the chemical reaction between aluminium and oxygen during the solidification. In the refining process, the high melting point oxide of Al_2_O_3_ (>2,000 °C) is unavoidably formed in traditionally metallurgical practices. If the AD technique is adopted in the final stage of the refining process, a certain amount of Al_2_O_3_ particles with diameters ≪10 μm should not float rapidly, according to the Stokes law, which dictates that the floating velocity is proportional to the square of the particle’s diameters. Thus, these small particles will be buried into melts. Owing to the weak wettability of Al_2_O_3_ particles during the solidification process, they would not only be inclined to agglomerate together but would also adsorb the surrounding S, Mn or other ions to form a larger OI (Fig. 1b) and become more buoyant. While the inter-dendritic microsegregation certainly enhances these adsorptions, their behaviours would also be chemically evidenced by our first-principles calculations. Owing to the strong electronic hybridizations among S and its nearest neighbours O and Al on the surface, α-Al_2_O_3_ favourably traps the free S ion and binds with Mn ion to initially nucleate the MnS-like cluster (Fig. 3a). As shown in Fig. 3b, the calculations also indicate that, as the number of S ions trapped by one absorbed Mn ion on the surface increases, the adsorption energies (Fig. 3b) and the binding energies (Fig. 3c) of the Mn-*n*S-like clusters weaken. Similar behaviours have been observed for the adsorption of additional Mn ions. The calculations highlight the following trend: as nucleated Mn+*n*S-like clusters coarsen to the crystalline phase on the surface, their interface binding energies with the substrate of Al_2_O_3_ weaken, which eventually causes their separation (see Supplementary Note 5 and Supplementary Figs 17–20). This situation is experimentally identified in the CS strips (see Supplementary Fig. 10).

According to the classical theoretical framework, the flow instability in the inter-dendritic region that is caused by the melt convection, which is faster than the movement of the solidification front, is the essential underlying mechanism of CS formation^4^. Our mesoscale phase-field simulations coupled with the fluid flow dynamics reveal that the inter-dendritic solute convection in Fe−C (0.36 wt.%) alloy is weak (typically ~10 μm s^−1^) and slower than the isotherm rate; therefore, no CS is expected to occur (see Supplementary Note 6 and Supplementary Figs 21–23). Once a large population of properly sized OIs is introduced, our simulations reveal the occurrence of CS, as shown in Fig. 3d,e. This finding is attributed to the sufficient volume fraction of the OI population that induces the flow instabilities in the mushy zone, as illustrated by the simulations of CS in Fe-C (0.36 wt.%) steels using the multiphase flow approach (see Supplementary Note 7). Figure 3d visualizes the simulated carbon distribution in the cavity within a unidirectional solidification, which demonstrates CS evolution. The simulated flow fields, the wavy isolines of solid fraction and the distribution of alumina in the cavity are demonstrated in Fig. 3e. The alumina concentrated along the CS strips is distinct.

Note that our simulations revealed that the appropriate size and population of OIs are two essential factors that affect CS formation. Within our current modelling, the most appropriate diameter of OIs, which is the first factor, has been determined to fall within the range of 5~30 μm. These OIs can efficiently enhance the local liquid flow, and alter its direction, which causes flow instability and the destabilization of the mushy zone. However, the size of OIs cannot be too small (that is, ≪5 μm) or large (that is, >30 μm). The results demonstrated that the OIs with diameters ≪5 μm float weakly and slowly to drift with the current of the thermosolutal convection or to be captured by solid, whereas the OIs with large diameters >30 μm rapidly float into the top bulk melt and their influences on the solidifying melt easily vanish. We found that this theoretical diameter range (5~30 μm; see Supplementary Note 7 and Supplementary Fig. 24) of OIs to induce CS formation corresponds with our experimentally observed scale (predominantly 5~50 μm) (see Supplementary Note 4). The discrepancies can be primarily attributed to the limitations of our modelling as we neglected the coagulation, growth and blocking of dendritic networks for OI. For the second factor, the modelling simulation also revealed that, as the population of the initial OIs with a diameter of 15 μm increases, the CS strips increase and become more significant (see Supplementary Fig. 25). At the adjacent onset site of the CS formation, the local volume fraction of OIs is estimated to be a minimum of 0.01% according to our simulations. Using the 3D X-ray tomography technique in Experiment **I** (see Supplementary Note 4 and Supplementary Figs 26,27), the volume fraction of OIs at the adjacent onset site of the CS formation is measured to be ~0.09%, which is within the same order of magnitude as our estimated data. Within the body of the CS region, the maximum measured volume fraction is 0.35%, which is one order magnitude larger than the adjacent onset site. This fact reflects the coagulation and growth of OIs during CS formation. These results convince us that the large volume fraction of the OI population with appropriate sizes and quantities induces the destabilization of the mushy zone and dominates CS formation.

## Discussion

**Figure 4:**
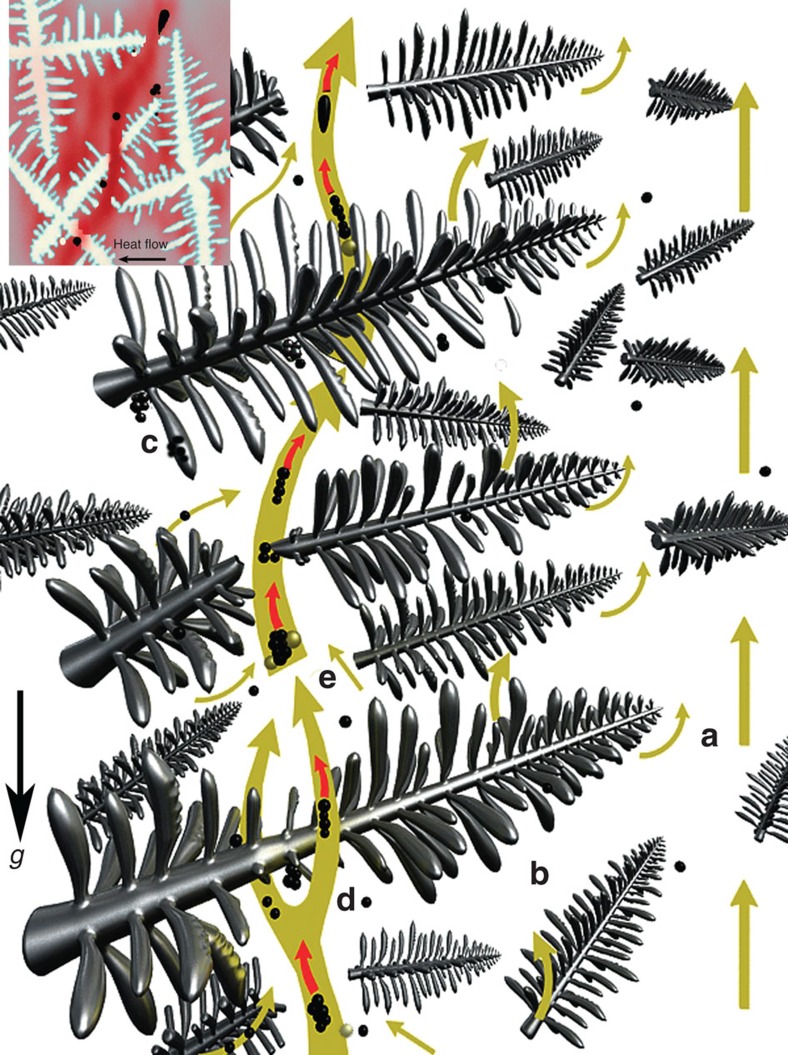
Schematic of inclusion flotation among the inter-dendritic regions. The schematic reveals the three-dimensional dendritic growth around the sidewall of the ingot. The inset shows the two-dimensional projection of the inter-dendritic regions. Five flotation modes have been highly suggested: (**a**) solute-enriched melts run along the primary dendrite into the bulk melt and float up in front of the dendrite tips; (**b**) solute-enriched melts vertically float across the dendrite trunks and branches; (**c**) the floating OIs are blocked by the dendrites; (**d**) together with the floating of accumulated OIs and minor amounts of bubbles, further re-melt, erode the dendrites to form an extra non-thermosolutal flow, and the flow channels are formed by the inclusion flotation and the introduction of its correlated solute-enriched melts; (**e**) the interfacial tension-driven flow coagulates and reshapes the clusters of OIs to strip-like chains. Solid spheres and yellow balls denote inclusion particles and gas bubbles, respectively. *g* denotes the gravity.

The OI flotation-driven CS occurrence has been neglected in long-term scientific and engineering practices. Thus, we defined this neglected flotation as a new driving force. During solidification, the fine OIs agglomerate and grow in multiple locations at a certain distance from the side wall of the ingot. These sufficient OIs in the mushy zones, which are featured by low solid fraction, will float up spontaneously, and, during their flotation, some OIs that are obstructed by dendrite arms will preferentially move incline-upwards due to the thermal field. Mechanically, the floating of the large population of OIs will effectively perturb the flow fields in the mushy zone and alter the local flow patterns, which destabilizes the mushy zone (see the wavy isolines of the solid fraction in Fig. 3e) and triggers the origin of the CS. As the destabilized mushy zone advances, the flotation of OIs not only drags the surrounding solute-rich melts to flow together but also causes the enrichment of adjacent OIs, low melting point impurities (that is, some sulphides) and bubbles. These retain the successive interactions with their surrounding melts and generate the macroscale CS occurrence, as illustrated in Fig. 4. Owing to the suck or adsorbed solute elements and sulphides (that is, MnS) around the OIs, they would lower the local melting point and re-melt or erode the dendritic trunks and branches, enrich and float up together, which promote the formation of CS. Despite the difficulty of dynamically observing this real-time process of CS formation, we trust that this mechanism is highly suggestive. In addition to this inclusion flotation, note that the interfacial tension-driven flow may serve a specific role in coagulating the impurity particles^24^ and reshaping the clusters of inclusions in the strip-like chains via the capillary force in streams.

It is worth noting that the previous established theories by experimental and numerical studies reveal that the thermosolutal convection is the essential factor that is responsible for the formation of CS in some superalloys^25^,^26^, aluminium alloys^1^, model alloys (Ga-25 wt.%In (ref. 11), Sn-5 wt.%Pb (ref. 27), Sn-20 wt.%Bi (ref. 28), and so on.) and even a few special steels (that is, high Si steels^29^). In these alloys, the high density contrast between the inter-dendritic melt and the bulk liquid is sufficient to drive intensive melt convection and produce CS (see Supplementary Fig. 28, Supplementary Note 7 and Supplementary Table 3). In the extensively applied steels, the concentration of the main element—carbon—is at least one order of magnitude less (typically, [C]≪0.77 wt.%) than these alloys; thus, the density contrasts in the melt cannot drive this strong convection. It is therefore difficult for the weak melt convection to induce the CS formation, but the OIs flotation does.

**Figure 5:**
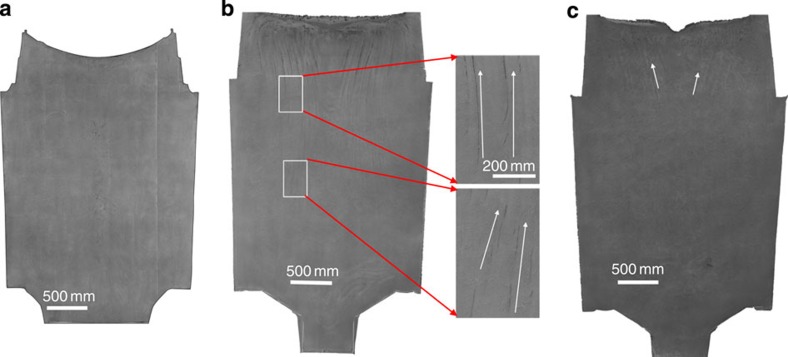
The fully sectioned and etched surfaces of three 100-ton ingots. These ingots consist of 30Cr2Ni4MoV steels with a diameter of 2.4 m and a height of 3.6 m. The arrows denote the CS. (**a**) **Experiment VI**: in the VCD technique, no CS is visualized. (**b**) **Experiment VII**: in the AD technique, the local slim CSs are indicated by arrows. (**c**) **Experiment VIII**: in the VCD technique, no CS has been observed in the ingot body, with the exception of two short slim CSs, as denoted by two arrows in the top feeder.

As a new mechanism for CS formation, it has to be examined in engineering practice. Three 100-ton industrial ingots (**VI**, **VII** and **VIII** in Table 1) with a diameter of 2.4 m and a height of 3.6 m have been produced by vacuum pouring, two ingots (**VI** and **VIII**) have been produced by the VCD technique and one ingot **VII** has been produced by the AD technique. As expected, almost no CS is observed in the fully dissected cross-section of Experiment **VI** (see, Fig. 5a), which corresponds to our current theoretical analysis due to the lower oxygen concentration with T.O=1.0 × 10^−3^ wt.%. In Experiment **VII**, the pre-heated and insulation feeder techniques have been adopted to reduce the centre-line porosity defects, while a small amount of Al is fed into the refining ladle to analyse the effect of oxygen concentration and OIs on the CS. The results reveal the presence of a few slim CSs enriched with Al_2_O_3_ (Fig. 5b and see Supplementary Movie 1) in the coarsened dendritic grain zones due to a slightly higher T.O=1.5 × 10^−3^ wt.%. This experiment validates the conclusion: oxygen is a source for initializing the CS in solidifying steels. By combining the techniques of Experiments **VI** and **VII**, avoiding the use of the AD technique and lowering the oxygen concentration to T.O=1.2 × 10^−3^ wt.%, the third 100-ton ingot (Experiment **VIII**) has been successfully produced. This ingot is perfect, as we expected. No any CS is detected in the body of the ingot, with the exception of two slim CSs in the top feeder (Fig. 5c and see Supplementary Movie 2). As listed in Supplementary Table 1, the mechanism has been re-confirmed in the large ingots (234 and 535 tons) by our collaboration partners of the heavy metal industries (see Supplementary Notes 2 and 3); it also matches the previously reported results for a 650-ton ingot^22^.

In traditional metallurgy, the CS in steels is primarily controlled in the solidification process via rapid cooling, mechanical vibration and electromagnetic stirring^5^,^6^, and so on. Increasing the cooling rate not only accelerates the freezing of liquid melt but also limits the flotation of OIs. The mechanical vibration and electromagnetic stirring externally alter the morphology of microstructures, which naturally affects the flow behaviour of the OIs. To some extent, these technologies may exert certain effects on the prohibition of CS but they exhibit intrinsic limitations to the applications in heavy steel ingots due to the thick thermal diffusion boundary layer and heavy bulk melts. Our current findings challenge the traditional approach to preventing CS formation from solidification control to upstream metallurgy control. Namely, via the low-oxygen purifying technology for maintaining a low level of T.O (typically, T.O ≪1.0 × 10^−3^ wt.%) and jointly limiting the other impurity elements (that is, S and P), the CS can be controlled in engineering practice. This purifying technology represents a significant breakthrough to pin the CS occurrence, which is operable and efficient for the industrial manufacture of steel ingots, slabs and castings. Conceptually, this approach comprises an innovative method in which the role of inclusion flotation highlights a new horizon and demonstrates a substantial difference from the traditionally recognized buoyancy force of natural convection. This investigation on the inclusion flotation reveals the underlying effect of oxygen on CS formation in steels and the importance of the control of oxygen concentrations in the steel industry, besides the already recognized manipulations of the elements C, Si, Mn, S and P.

## Methods

### Experiments of ingots

Five experimental steel ingots (500 kg carbon steels (**Experiments I, II, III, and V)** and an alloying steel (**Experiment IV**) have been synthesized with a sand mould. (1) The following chemical compositions (measured, wt.%) are utilized in **Experiment I**: C 0.47, Si 0.26, Mn 0.54, S 0.016, P 0.020, T.O 0.0056 and Fe balanced. The steel was melted at 1,600 °C by an induction furnace and poured at 1,550 °C in the atmosphere after the Al deoxidation (AD) process. (2) The following chemical compositions are utilized in **Experiment II**: C 0.47, Si 0.44, Mn 0.49, S 0.005, P 0.005, T.O 0.001 and Fe balanced. It was refined and poured in vacuum conditions with both Al-free and vacuum carbon deoxidation (VCD) techniques. (3) The following chemical compositions are utilized in **Experiment III**: C 0.44, Si 0.51, Mn 0.68, S 0.013, P 0.006, T.O 0.0015 and Fe balanced. The experimental methods are similar to the methods employed in **Experiment II**. 4) The following chemical compositions are utilized in **Experiment IV**: C 0.07, Si 1.34, Mn 2.32, Cr 9.62, W 1.52, V 0.25, S 0.005, P 0.007, T.O 0.0020 and Fe balanced. The experimental methods are similar to the methods employed in **Experiment II**. (5) The following chemical compositions are utilized in **Experiment V**: C 0.45, Si 0.54, Mn 0.64, S 0.008, P 0.009, T.O 0.0007 and Fe balanced. The VCD technique has been applied in the melting and refining stages of the molten steel while no Al was added. After the refining process, the level of oxygen concentration at T.O=0.0007 has been yielded in the melt and argon has been subsequently pumped into the furnace chamber to increase the maximum pressure to one atmosphere for pouring; it was poured at 1,550 °C. For these five ingots, the sand mould was used to produce a 500 kg round ingot. The as-cast ingot was cut in half along the longitudinal axis. After the ingot was grinded, polished and etched by a 20% HNO_3_-5%H_2_SO_4_-H_2_O solution, a 20% HNO_3_-H_2_O solution and a 5% HNO_3_-H_2_O solution, respectively, CS was observed. The inclusions in samples cut from the CS zone were observed and identified by scanning electron microscopy (SEM) and energy dispersive spectroscopy (EDS).

Three 100-ton 30Cr2Ni4MoV ingots (**Experiments VI**, **VII** and **VIII**) have been experimentally synthesized with a cast iron mould. (1) The following chemical compositions (measured, wt.%) are utilized in **Experiment VI**: C 0.22, Si 0.01, Mn 0.13, Cr 1.7, Ni 3.4, Mo 0.30, V 0.086, S 0.005, P 0.006, T.O 0.001 and Fe balanced. (2) The following chemical compositions are utilized in **Experiment VII**: C 0.22, Si 0.07, Mn 0.06, Cr 1.6, Ni 3.4, Mo 0.28, V 0.08, S 0.002, P 0.005, T.O 0.0015 and Fe balanced. (3) The following chemical compositions are utilized in **Experiment VII**: C 0.22, Si 0.01, Mn 0.06, Cr 1.67, Ni 3.6, Mo 0.27, V 0.08, S 0.003, P 0.005, T.O 0.0012 and Fe balanced. The three ingots **VI**, **VII** and **VIII** have been produced by the following processes: electric arc furnace-ladle furnace-vacuum degassing and mould stream degassing. The vacuum pouring temperature is controlled to 1,575 °C. Note that, in both **VI** and **VIII**, the VCD technique was adopted; however, the AD technique was adopted for **VII**, and 0.014 wt.% Al was fed in the ladle furnace to reduce the oxygen concentration. Subsequently, the three as-cast ingots were cut in half along the longitudinal axis. After the ingot sections were grinded, polished and etched by a 20% HNO_3_-5%H_2_SO_4_-H_2_O solution, a 20%HNO_3_-H_2_O solution and a 5% HNO_3_-H_2_O solution, respectively, the CS was examined (see Supplementary Movie 3). The experiments demonstrated that the maximum measured solidification times are 27 h for **VI** and 32 h for **VII** and **VIII**.

### 3D X-ray characterisations

The volume fraction of OIs in the CS in the steel ingot of **Experiment I** has been measured using the 3D high-resolution transmission X-ray tomography (HRTXRT) technique with the lab-based Xradia Versa XRM-500 system. X-ray tomography imaging was performed for the cylindrical (Φ3 × 25 mm) or rectangular (4 × 4 × 35 mm) specimens that were cut from the CS region. The working accelerating voltage was 140 kV. A total of 1,600 images, which were each exposed for 4 s, were recorded as the sample was rotated by 360° and computationally reconstructed via a filtered back projection algorithm to produce a 3D image with a voxel size of ~3.5 μm. The equivalent volume diameter method was employed to obtain the dimensions of OIs.

### First-principles calculations

The modelling simulations have been performed using the Vienna Ab initio Simulation Package (VASP)^30^ within the framework of the density functional theory, based on the plane-wave method. We adopted the generalized-gradient-approximation within the Perdew-Burke-Ernzerhof (PBE) parameterization scheme for the exchange-correlation function. Brillouin zone integrations were performed for the *k*-mesh 5 × 5 × 1 according to the Monkhorst and Pack technique. The energy cutoff for the plane-wave expansion of the eigenfunctions was set to 400 eV. The optimization of the structural parameters was achieved by the minimization of the forces and stress tensors. The simulation primarily focuses on the interactions among the S atom, the Mn+*n*S atom complex and the surface of the solid alumina, which frequently appears in the steel melt. To establish the modelling system, we made two assumptions: (1) that the element S is considered to be the S atom or the Mn+*n*S atom complex in the melt and (2) the interfacial interactions among the alumina, S atom and Mn+*n*S atom complex with the iron melt is disregarded. We have selected α-alumina (α-Al_2_O_3_) as the modelling structure. The optimized lattice constants, the enthalpy of formation [*ΔH*=−153.99 kJ (mole of atoms)^−1^], the bulk band gap (*E*_g_=5.9 eV at the Γ-point) of the bulk α-Al_2_O_3_ corresponds with the previously reported results. Along the [0001] direction, the stacking sequence can be viewed as O-Al-Al-O-Al-Al-O. The distance between any two adjacent O layers is ~2.2 Å in the bulk equilibrium phase. Therefore, three (0001) surface terminations are currently available, namely, the Al-, AlO- and O-terminated surfaces. Consistent with the reported results, the clean AlO-terminated (0001) surface has been energetically most favourable result with the lowest surface energy of 1.68 J m^−2^. Note that our calculations have been based on the non-dipole AlO-terminated surface, which denotes a single outermost Al-layer and a sub-outermost O-layer. The optimized surface structure indicates that the space distance between the topmost layer and the second layer is significantly reduced (Δ*l*≈0.738 Å being ~78% of the unrelaxed distance). These results show that the topmost Al atom is almost in the same layer as the second oxygen layer. This situation is attributed to the finding that the coordination number of the topmost Al atom is much less than the crystal. To simulate the adsorption on the surface of α-Al_2_O_3_, we constructed a slab with an 18-layer-thickness, a 15 Å vacuum depth along the [0001] direction, and a surface unit cell with a 2 × 2 dimension. For all surface calculations, the bottom nine layers have been kept frozen; the other nine layers have been allowed to relax.

### Macro/meso-scale simulations

The theoretical model of multiphase flow, which considers the effect of the melt on the particles and the particles on the melt flow distribution, is incorporated into the macrosegregation model within the classical theoretical framework. The discrete phase model^31^ is employed to calculate the flotation of particles, in which the trajectories of the particles that move in the flow field have been computed using a Lagrangian approach. The particle-fluid interactions are considered by encompassing the effect of the melts on the particle trajectories and the effect of the particles on the melts flow distribution. Details of the Lagrangian modelling approach for the treatment of particle motion have been reported elsewhere^31^. The formulations of the macrosegregation model are provided in Supplementary Note 7. In addition, all particles are assumed to exhibit a spherical shape. Before solidification, the liquid is stationary and particles are randomly injected into the melt in space. The two types of simulated solidification process are sketched in Supplementary Fig. 29. The density of the molten carbon steel Fe-0.36 wt.% C (*ρ*_l_) and the Al_2_O_3_ particles (*ρ*_p_) are 6.99 and 3.64 g cm^−3^, respectively. The dynamic viscosity of the steel melt *μ* is equivalent to 0.0042 Pa·s.

To assess the solute convection in advance of the dendritic solidification front, the quantitative two-dimensional Karma phase-field model for the binary alloy solidification^32^ in the coupling of fluid flow dynamics has been adopted to investigate the evolution of the solid-liquid interface in the condition of melt flow. In this model of the crystallization, the evolution of the phase state has been described as a function of the order parameter ψ, the composition *C* and the temperature *T*. The ordering parameter ψ has a constant value in the solid and liquid phases and varies smoothly across the thin diffuse solid-liquid interface. The transport of solute in liquid has been induced not only by diffusion but also by convection. Assuming that the fluid is incompressible, the Navier–Stokes equations have been considered to describe the fluid motion. The gravity-driven natural convection induced by solute and thermal expansion has been considered via a source term in the viscous equation using a Boussinesq approximation^8^. To depict the evolution of thermosolutal convection in the regions between columnar dendrites, a conventional upward directional solidification of Fe–0.36 wt.% C alloy has been employed with several different processing parameters. The calculations have been performed on a 2,117 × 6,880 μm^2^ rectangular grid via an adaptive mesh and parallel computing procedure. A vertical temperature gradient of 37 °C cm^−1^ has been established, in which the top is hotter than the bottom, and the isotherm rate varies from 13.5 to 1,081.1 μm s^−1^. Details of the model and numerical implementations are provided in Supplementary Note 6. The computational parameters employed in the numerical simulations are listed in Supplementary Table 4.

## Author contributions

D.L. proposed the effect of OIs on channel segregation. P.F., H.L. and Y.Lu. performed the ingot casting, compositions analysis and observation of channel segregation. X.M. performed the inclusion measurements and analyses in the channel segregate regions. X.-Q.C. performed the density functional theory calculations. Y.Ch. performed the mesoscale simulations and Y.Ca. performed the process scale simulations of OI-induced CS formation. Y.Li. proposed the original problem and supervised the investigation. D.L. and X.-Q.C. wrote the paper with assistance from all authors. All authors contributed to the discussions in the manuscript.

## Additional information

**How to cite this article:** Li, D. *et al.* Inclusion flotation-driven channel segregation in solidifying steels. *Nat. Commun.* 5:5572 doi: 10.1038/ncomms6572 (2014).

## Supplementary Material

Supplementary Figures, Tables, Notes, Methods and References Supplementary Figures 1-29, Supplementary Tables 1-4, Supplementary Notes 1-7, Supplementary Methods and Supplementary References.

Supplementary Movie 1 The animation of the fully sectioned and etched surfaces of the 100-ton ingot (experimental-VII in the main text)

Supplementary Movie 2 The animation of the fully sectioned and etched surfaces of the 100-ton ingot (experimental-VIII in the main text)

Supplementary Movie 3 The animation of casting, finishing, sectioning, and etching details of 100-ton ingots.

## Figures and Tables

**Table 1:** Operating conditions and results of eight designed experiments (We have summarised a total of 18 dissected ingots, which are weighted from 0.5 to 650 tons in Supplementary Table 1).

**Expt.**	**Condition**			**Result**				
	**Mass (ton)**	**Pouring Method**	**Deoxidation**	**T.O (10**^−3^ **wt.%)**	**C (wt.%)**	**S (wt.%)**	**P (wt.%)**	**CS**
**I**	0.5	Air	AD	5.6	0.47	0.016	0.020	Yes
**II**	0.5	Vac.	VCD	1.0	0.47	0.005	0.005	No
**III**	0.5	Vac.	VCD	1.5	0.44	0.013	0.006	Yes**
**IV**	0.5	Vac.	VCD	2.0	0.07	0.005	0.007	Yes*
**V**	0.5	Air	VCD	0.7	0.45	0.008	0.009	No
**VI**	100	Vac.	VCD	1.0	0.22	0.005	0.006	No
**VII**	100	Vac.	AD	1.5	0.22	0.002	0.005	Yes*
**VIII**	100	Vac.	VCD	1.2	0.22	0.003	0.005	No

Here, * denotes slight CS and ** denotes very slight CS.

The AD and VCD techniques denote the aluminium deoxidation and the vacuum carbon deoxidation, respectively. T.O, C, S and P represent the average total oxygen concentration, carbon concentration, sulphur concentration and phosphorus concentration, respectively.
