# Supplementary material for: Inclusion flotation-driven channel segregation in solidifying steels
Source: Nat Commun. 2014 Nov 25;5:5572. doi: 10.1038/ncomms6572 (PMC4263320; doi:10.1038/ncomms6572)
Supplement: Supplementary Figures, Tables, Notes, Methods and References — Supplementary Figures 1-29, Supplementary Tables 1-4, Supplementary Notes 1-7, Supplementary Methods and Supplementary References. [file ncomms6572-s1.pdf]

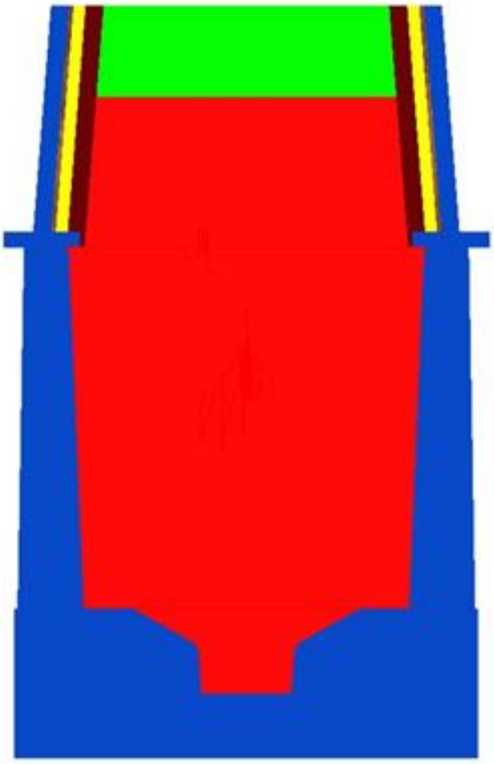

Supplementary Figure 1 | The section sketch of the 100-ton ingot used in simulations[1]. The blue, red, yellow (including dark brown), and green denote the mould, ingot, insulation and covering flux, respectively.

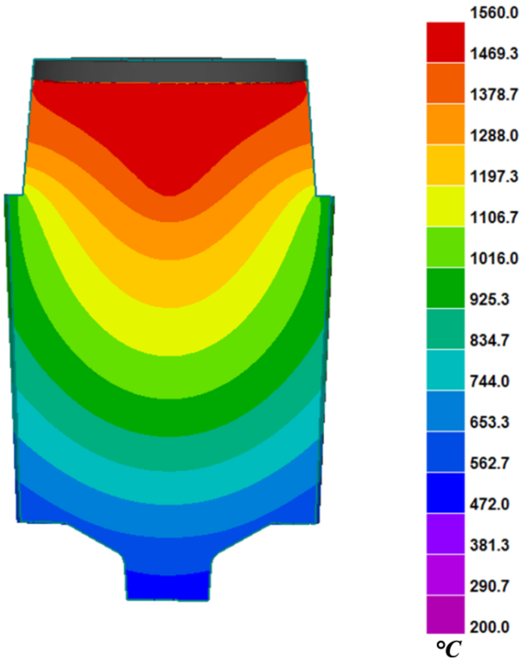

Supplementary Figure 2 | The simulated temperature field after full solidification of the ingot body.

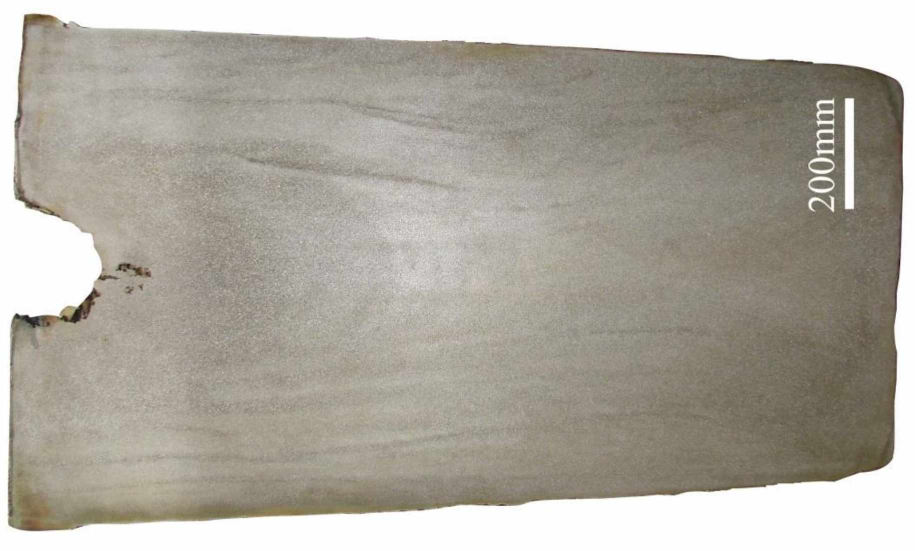

Supplementary Figure 3 | 45C 5-ton bottom poured ingot (Ingot IX in Supplementary Table 1). The fully sectioned surface of 5-ton 45C etched ingot (Experiment IX of C 0.49, Si 0.69, Mn 0.77, S 0.018, P 0.026, T.O 0.0036 and Fe balanced in its chemical compositions (wt.%)). The steel was melted at 1600 °C by induction furnace, and poured in the sand mould at about 1550 °C in the atmosphere after the Al deoxidation (AD) process. The as-cast ingot was cut in half along the longitudinal axis. After the ingot was grinded, polished and etched by the 20% HNO_3-5%H_2SO_4-H_2O solution, the 20%HNO_3-H_2O solution, and the 5%HNO_3-H_2O solution, respectively, the channel segregation (CS) was examined. The results demonstrated that there was serious CS in the ingot.

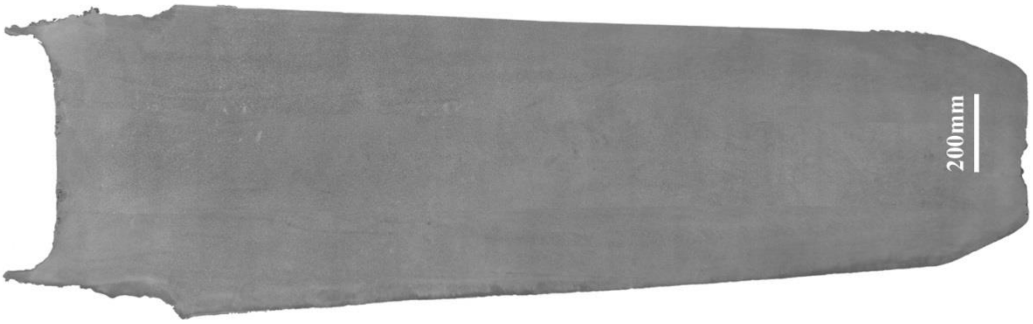

Supplementary Figure 4 | 45CrMoV 5.8-ton bottom poured ingot (Ingot X in Supplementary Table 1). The fully sectioned surface of 5.8-ton 45CrMoV etched ingot (Experiment X of C 0.45, Si 0.24, Mn 0.76, S 0.003, P 0.010, Cr 0.61, Mo 0.11,V 0.10, T.O 0.0007 and Fe balanced in its chemical compositions (wt.%)). The pouring temperature was 1550 °C and the die mould temperature was 80 °C. The ingot was fabricated by the electric arc furnace-ladle furnace-vacuum degassing and bottom filling in the atmosphere. To reduce quickly the concentration of dissolved oxygen in the melt, 0.085% Al was added into the melt in the refining stage of the ladle furnace via the AD technique. The as-cast ingot was cut in half along the longitudinal axis. After the ingot was grinded, polished and etched by the 20%HNO_3-5%H_2SO_4-H_2O solution, the 20%HNO_3-H_2O solution, and the 5%HNO_3-H_2O solution, respectively, the CS was examined. The results demonstrated that there were only very slight CSs in the ingot.

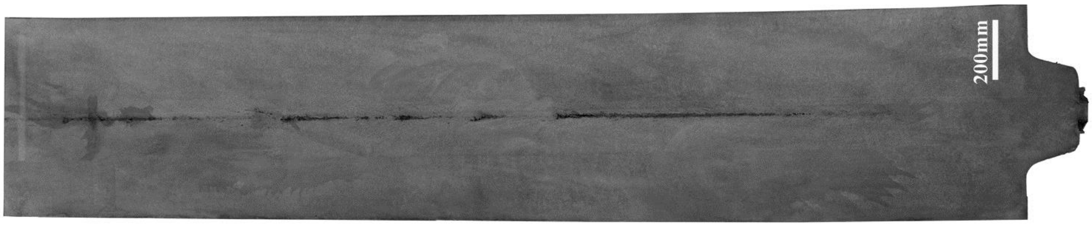

Supplementary Figure 5 | 42CrMo 14-ton bottom poured ingot (Ingot XI in Supplementary Table 1). The fully sectioned surface of 14-ton 42CrMo etched ingot (Experiment **XI** of C 0.41, Si 0.27, Mn 0.81, S 0.003, P 0.009, Cr 1.15, Mo 0.25,V 0.10, T.O 0.001 and Fe balanced in its chemical compositions (wt.%)). The pouring temperature was 1550 °C and the die mould temperature was 80 °C. In addition, the ingot was produced by the electric arc furnace-ladle furnace-vacuum degassing and bottom filling in the atmosphere. To reduce the content of the dissolved oxygen in the melt, 0.017% Al was added into the melt in the refining stage of ladle furnace via the AD technique. The as-cast ingot was cut in half along the longitudinal axis and its feeder was removed. After the ingot was grinded, polished and etched by the 20% HNO_3-5%H_2SO_4-H_2O solution, the 20%HNO_3-H_2O solution, and the 5%HNO_3-H_2O solution, respectively, there has been no CS found in the ingot body, except for the occurrence of the centreline porosities, as visualized by the dark line.

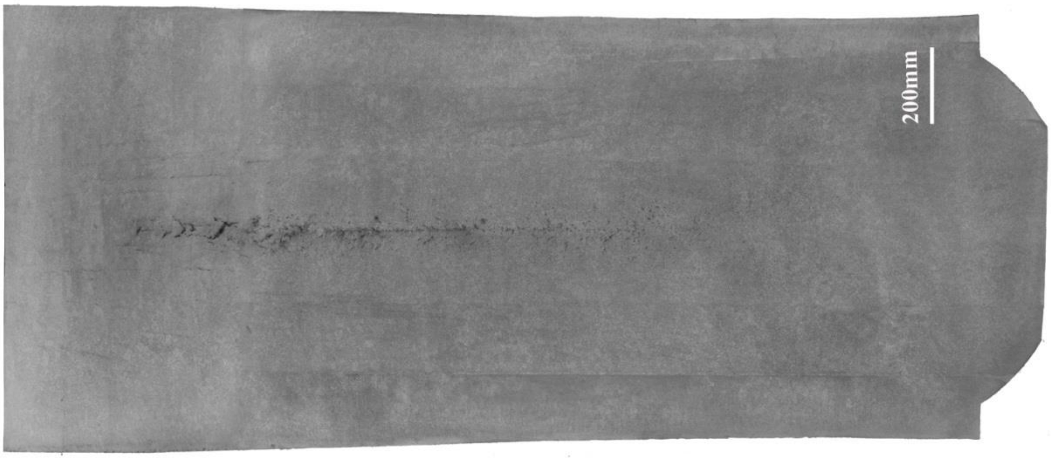

Supplementary Figure 6 | 42CrMo 16-ton bottom poured ingot (Ingot XII in Supplementary Table 1). The fully sectioned surface of 16-ton 42CrMo etched ingot (Experiment **XII** of C 0.42, Si 0.34, Mn 0.82, S 0.002, P 0.015, Cr 1.12, Mo 0.22,V 0.08, T.O 0.0008 and Fe balanced in its chemical compositions (wt.%)). The pouring temperature was 1550 °C and the die mould temperature was 80 °C. The ingot was produced by the electric arc furnace-ladle furnace-vacuum degassing and bottom filling in the atmosphere. To reduce the content of dissolved oxygen in the melt, 0.01% Al was added into the melt in the refining stage of ladle furnace via the AD technique. The as-cast ingot was cut in half along the longitudinal axis and its feeder was removed. After the ingot was grinded, polished and etched by the 20% HNO_3-5%H_2SO_4-H_2O solution, the 20%HNO_3-H_2O solution, and the 5%HNO_3-H_2O solution, respectively, only some slight CSs have been observed in the ingot body but the centreline porosity defect was obviously seen.

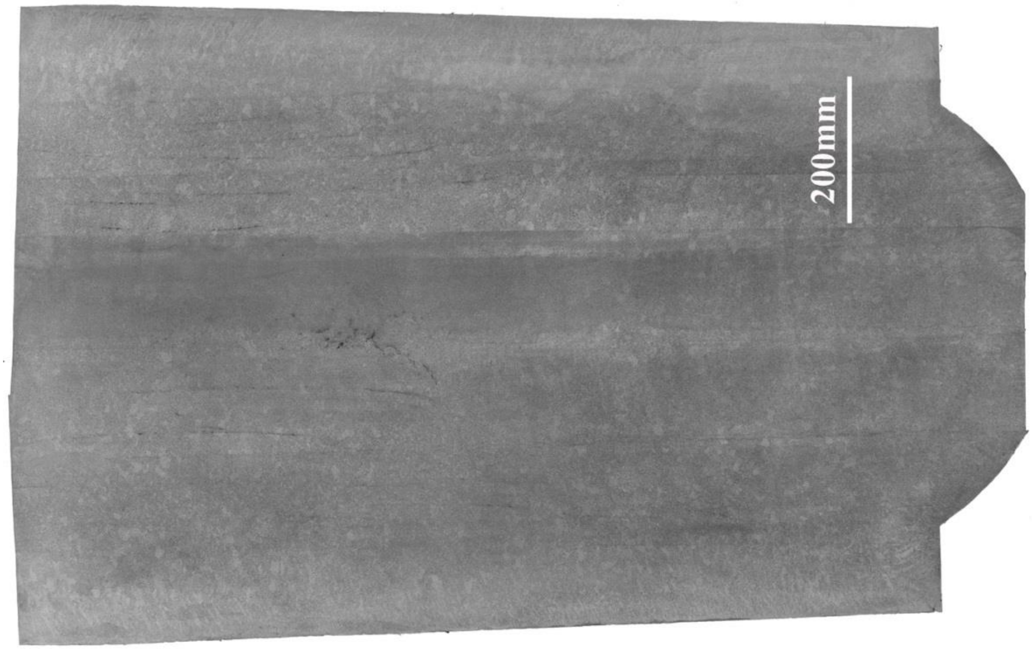

Supplementary Figure 7 | 42CrMo 16-ton bottom poured ingot (Ingot XIII in Supplementary Table 1). The fully sectioned surface of 16-ton 42CrMo etched ingot (Experiment **XIII** of C 0.45, Si 0.29, Mn 0.77, S 0.002, P 0.008, Cr 1.08, Mo 0.22,V 0.081, T.O 0.0011 and Fe balanced in its chemical composition (wt.%)). Compared with the Ingot **XII**, we changed the pouring configuration with different designs of the die mould. The pouring temperature was 1550 °C and the die mould temperature was 80 °C. The as-cast ingot was produced by the electric arc furnace-ladle furnace-vacuum degassing and bottom filling in the atmosphere. To reduce the content of dissolved oxygen in the melt, 0.01% Al was added into the melt in the refining stage of ladle furnace via the AD technique. The ingot was cut in half along the longitudinal axis and its feeder was removed. After the ingot was grinded, polished and etched by the 20%HNO_3-5%H_2SO_4-H_2O solution, the 20%HNO_3-H_2O solution, and the 5%HNO_3-H_2O solution, respectively, only some slight CSs existed in the ingot body while the centreline porosity defect can be observed.

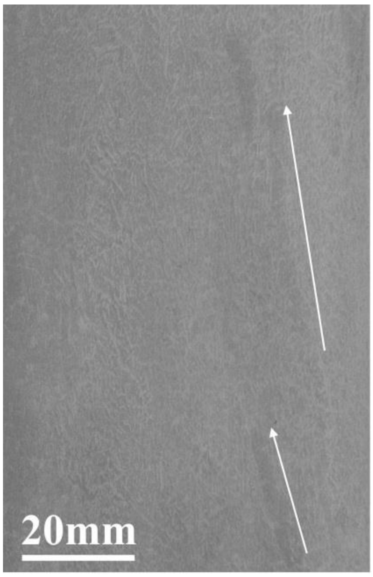

Supplementary Figure 8 | 34CrNiMo6 20-ton bottom poured ingot (Ingot XIV in Supplementary Table 1). The sectioned surface of etched sample taken from 20-ton 34CrNiMo6 ingot (Experiment **XIV** (the forging crankshaft using a 20-ton ingot) of C 0.35, Si 0.34, Mn 0.74,Cr 1.46, Ni 1.58, Mo 0.19, S 0.004, P 0.005, T.O 0.0010 and Fe balanced in its chemical compositions (wt.%)). The ingot was produced by the electric arc furnace-ladle furnace-vacuum degassing and the AD technique as well as bottom filling in the atmosphere. The measured sample was taken from the crankshaft body after the forging and heat-treatment processes. The sample was grinded, polished and etched by the 20%HNO_3-5%H_2SO_4-H_2O solution, the 20%HNO_3-H_2O solution, and the 5%HNO_3-H_2O solution, respectively, there existed very slight CSs in the sample as shown by the arrows.

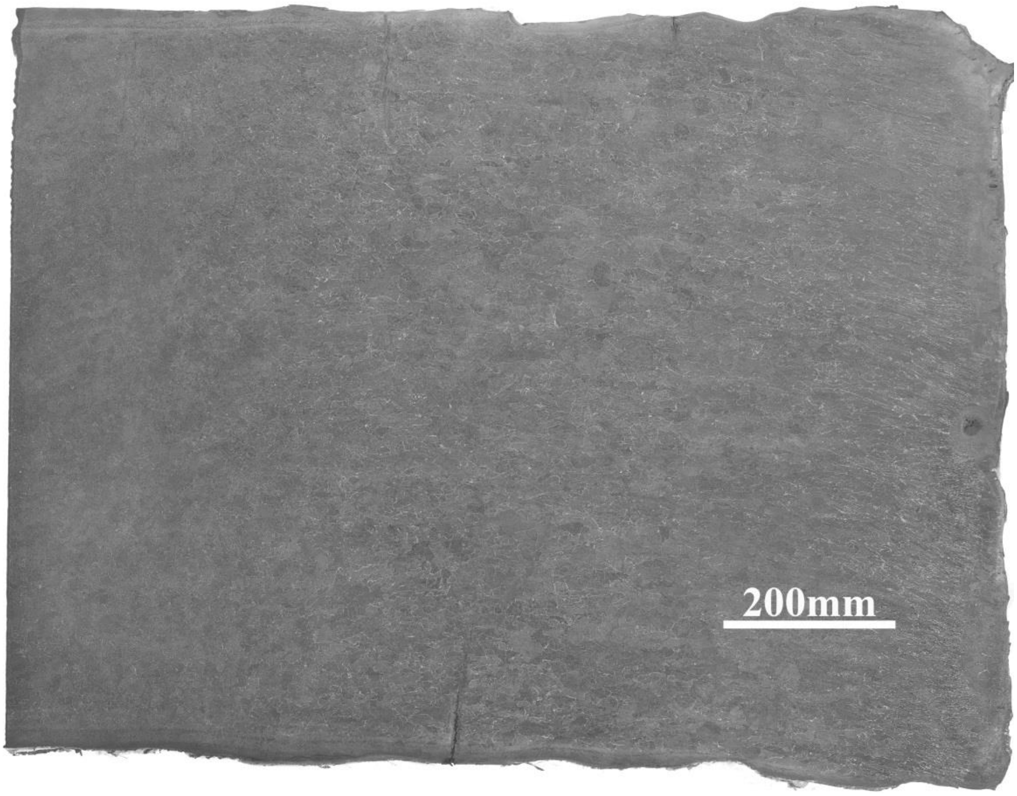

Supplementary Figure 9 | 12Cr2Mo1 69-ton top poured ingot (Ingot XV in Supplementary Table 1). The sectioned surface of 69-ton 12Cr2Mo1 etched ingot (Experiment XV of C 0.147, Si 0.051, Mn 0.33, S 0.005, P 0.010, Cr 2.24, Mo 0.890, T.O 0.0015 and Fe balanced in its chemical compositions (wt.%)). The pouring temperature was 1570 °C and the die mould temperature was 80 °C. The ingot was produced by electric arc furnace-ladle furnace-vacuum degassing-mould stream degassing and the vacuum carbon deoxidation (VCD) technique was adopted. The diameter of the ingot was 1727 mm, and the experimental sample with a length of 1200 mm and a width of 1000 mm was located at upper part of the ingot body and away from the hot top about 200 mm. The sample was cut in half along the longitudinal axis. After the sample was grinded, polished and etched by the 20%HNO_3-5%H_2SO_4-H_2O solution, the 20%HNO_3-H_2O solution, and the 5%HNO_3-H_2O solution, respectively, the results showed no CS in the sample.

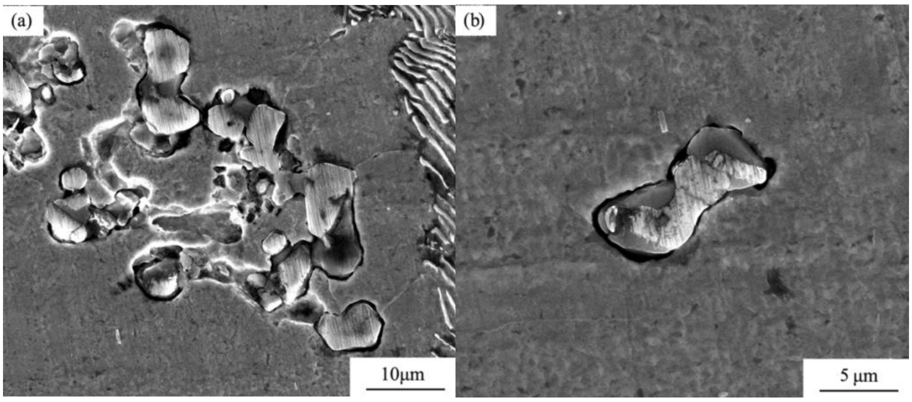

Supplementary Figure 10 | The morphologies of alumina in the CS zones. (a) the cluster-like alumina and (b) the separated alumina.

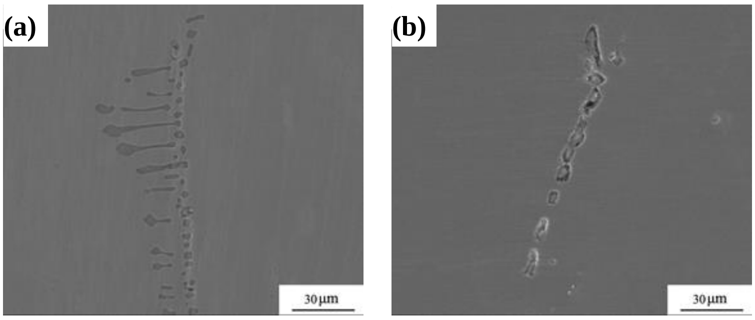

Supplementary Figure 11 | The large-size MnS inclusions in the CS zones. (a) The strip-like MnS and (b) the strip-like skeletal MnS.

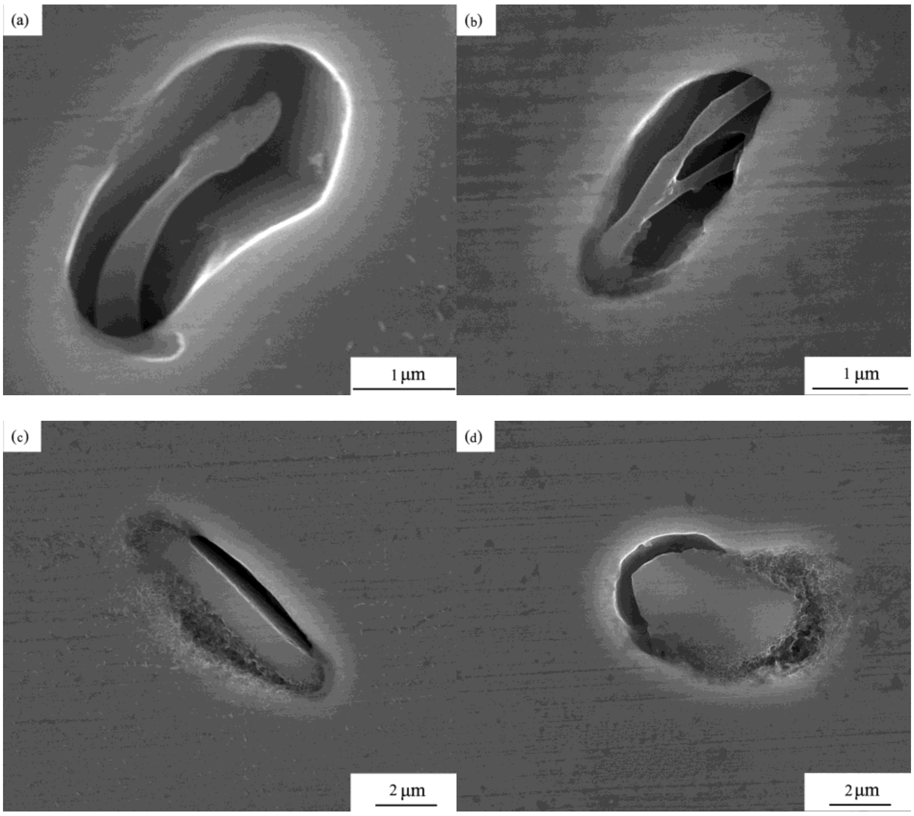

Supplementary Figure 12 | The small-size MnS inclusions in the CS zones. (a) and (b) the dendritic MnS. (c) The acetabular MnS and (d) the granular MnS.

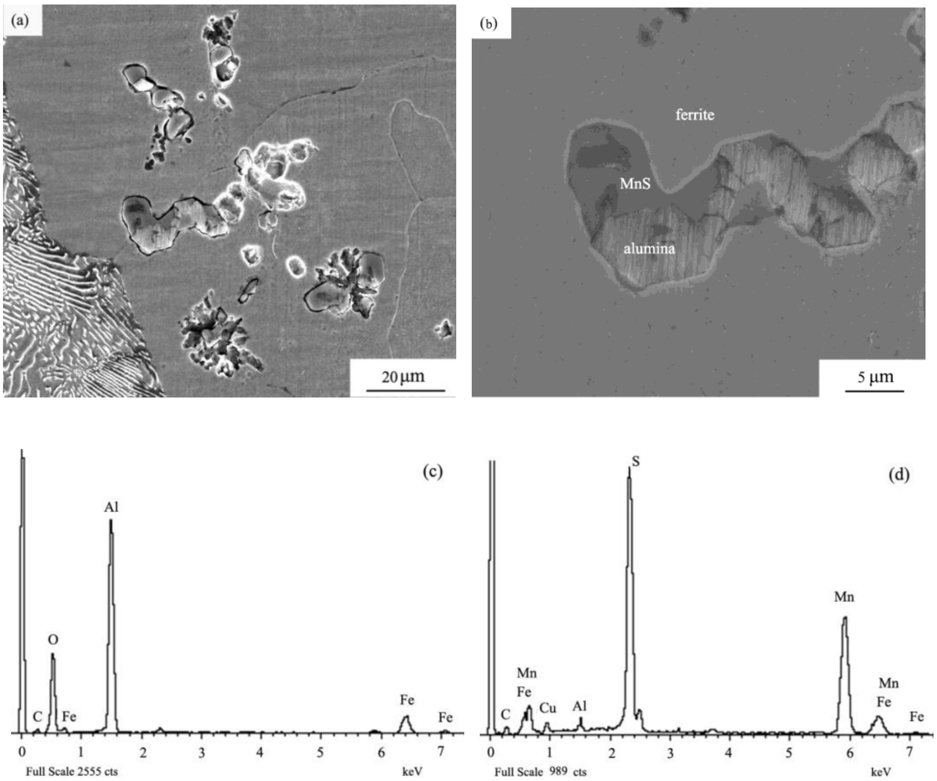

Supplementary Figure 13 | The coexistence of MnS and cluster-like alumina. (a) The coexistence of cluster-like alumina and MnS. (b) Zoom-in image of (a) showing coexistence phenomenon. (c) The EDS result for alumina in (b). (d) The EDS result for MnS in (b).

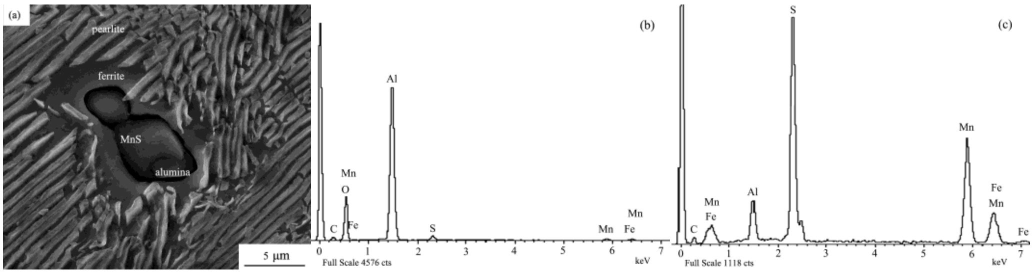

Supplementary Figure 14 | The coexistence of MnS and alumina. (a) The coexistence of separate alumina and MnS. (b) The EDS result for alumina zone in (a). (c) The EDS result for MnS zone in (a).

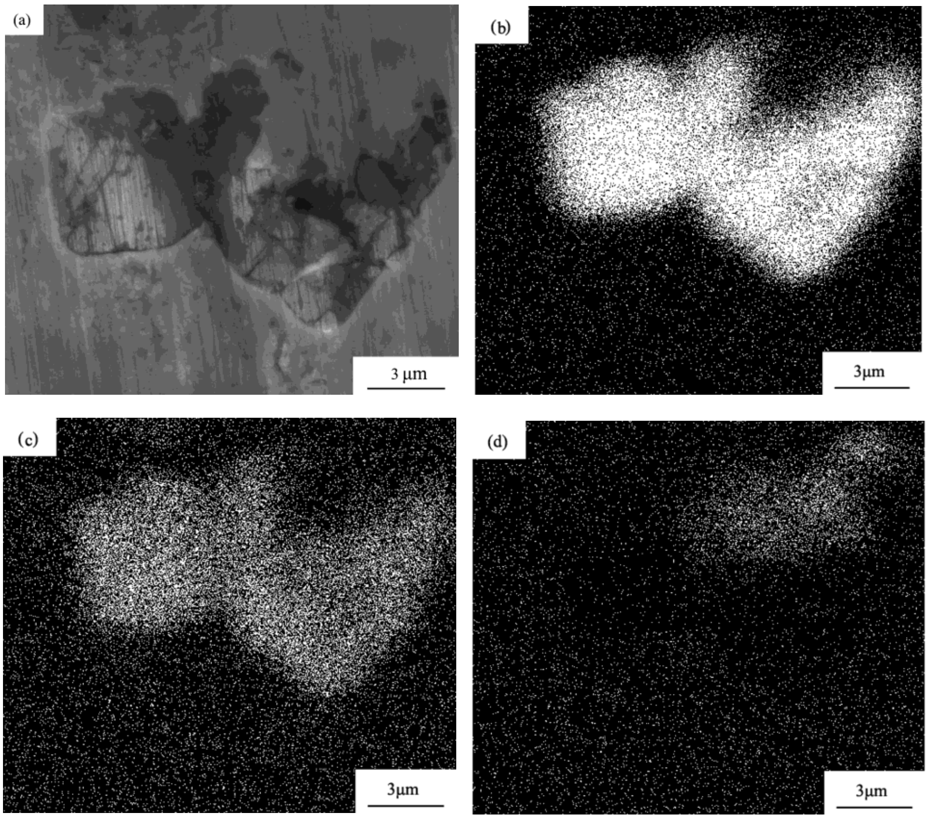

Supplementary Figure 15 | The enrichment of S content near alumina. (a) The SEM image of alumina. (b) The aluminium element area distribution image by EDS mapping. (c) The oxygen element area distribution image by EDS mapping. (d) The sulfur element area distribution image by EDS mapping.

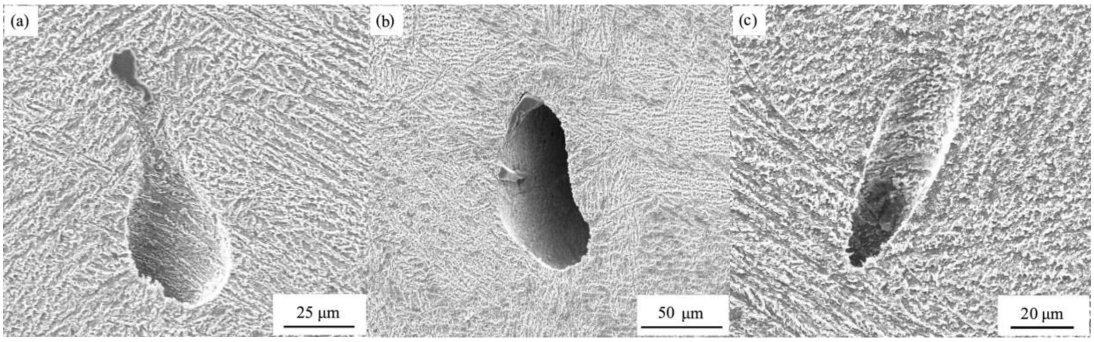

Supplementary Figure 16 | The bubble-like cavities in CS. (a), (b) and (c) show three typical morphologies of bubble-like cavities at different positions of CS.

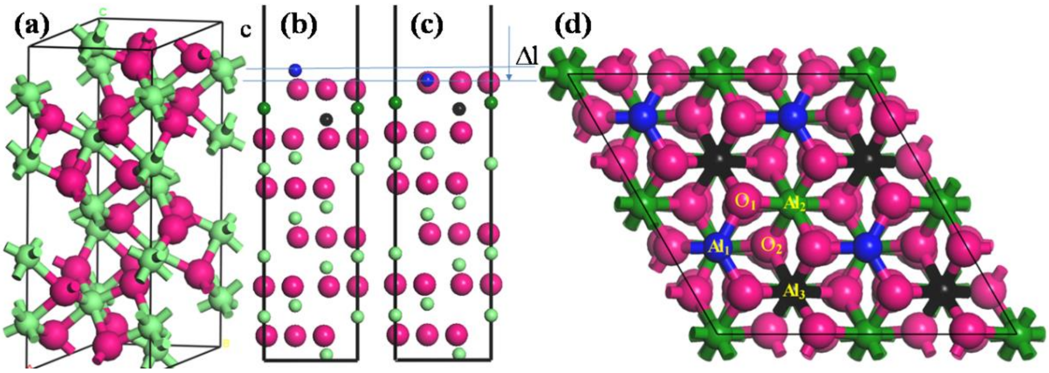

Supplementary Figure 17 | Structural modelling of first-principles calculations. (a) The lattice structure of α-Al_2O_3 in its conventional hexagonal unit cell (space group No. 167 ($R\bar{3}c$ hexagonal axes) with the optimized (experimental) lattice constants a = 4.81052 (4.795) Å and c = 13.1257 (13.095) Å with Al at 12cWyckoff site (0, 0, 0.35222 (0.35229)) and O at 18e Wyckoff site (0.3061 (0.3062), 0, 1/4). The experimental data refers to Supplementary reference [2]. (b) The unrelaxed AlO-terminated clean (0001) surface. (c) The relaxed AlO-terminated clean (0001) surface. (d) The projected two-dimensional AlO-terminated (0001) surface with the 2 × 2 unit cell. Large (red) balls and small (green, blue and black) balls denote oxygen and aluminium atoms, respectively.

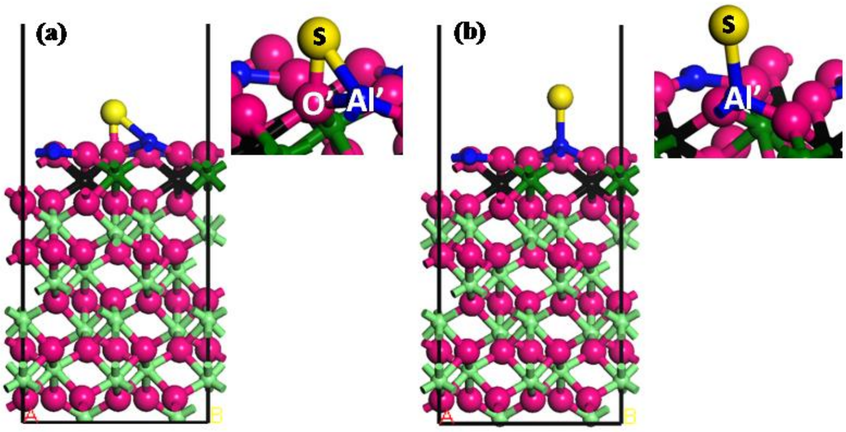

Supplementary Figure 18 | Individual S adsorption sites on the surface of α-Al_2O_3. (a) The most stable S trapped site in which S is bounded to both its nearest neighboring oxygen and Al atoms with a stable adsorption energy of −2.0 eV. (b) The stable S adsorption site in which S is only bounded to its nearest neighboring Al atom with a less stable adsorption energy of −0.82 eV. The upper right panels are the enlarged figures around the S adsorption sites in panels (a) and (b) to clearly show the local structural details. Additionally, the nearest neighboring O and Al atoms of the trapped S atom are marked by O’ and Al’, respectively.

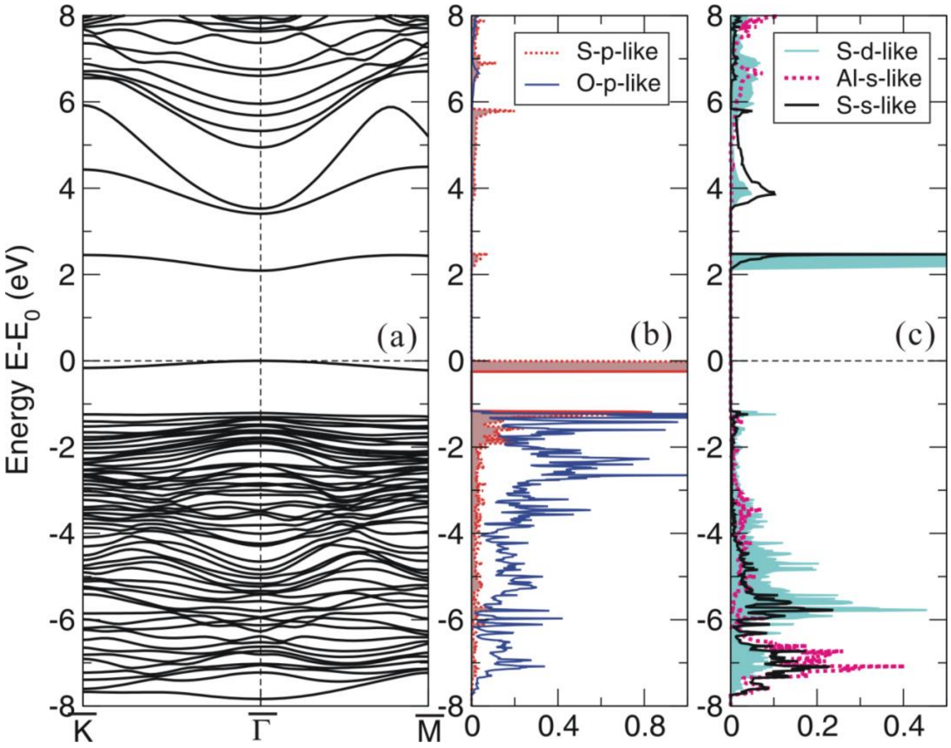

Supplementary Figure 19 | Electronic structure. (a) The electronic structure of the most stable S adsorption on the surface of α-Al_2O_3 [as shown in Figure 3a in the main text]. (b) and (c) the calculated projected densities of states (s-like, p-like and d-like DOSs) of the trapped S atoms and its two nearest neighboring Al and O atoms. The top of the valence band has been set to the energy of zero.

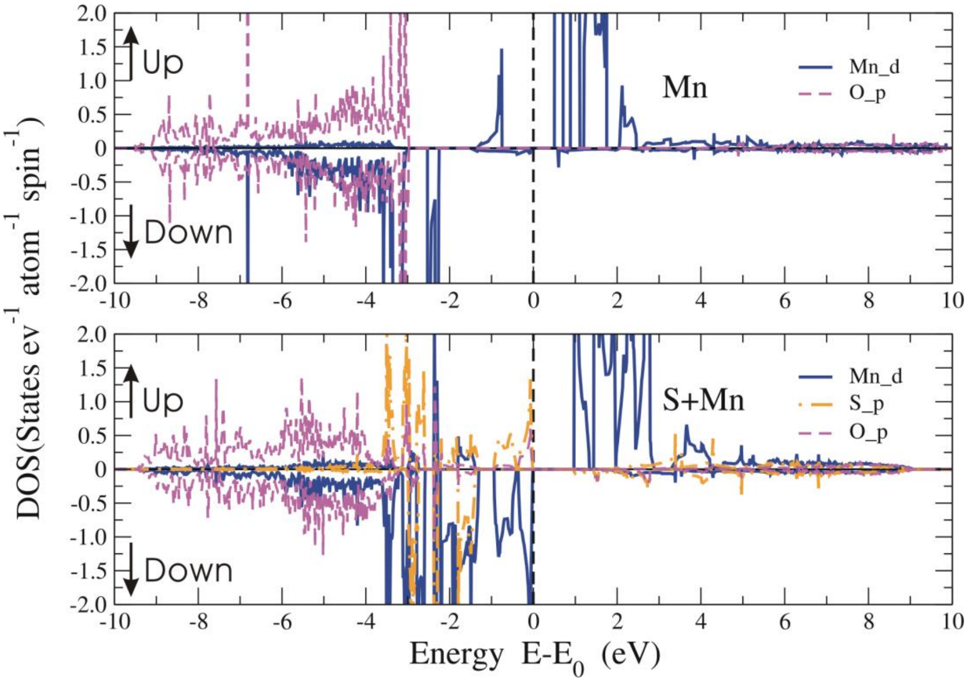

Supplementary Figure 20 | The electronic structure of the most stable Mn and Mn + S adsorptions on the surface of α-Al_2O_3. The top of the valence band has been set to the zero energy. Up and down arrows denote the spin-up and spin-down projected densities, respectively.

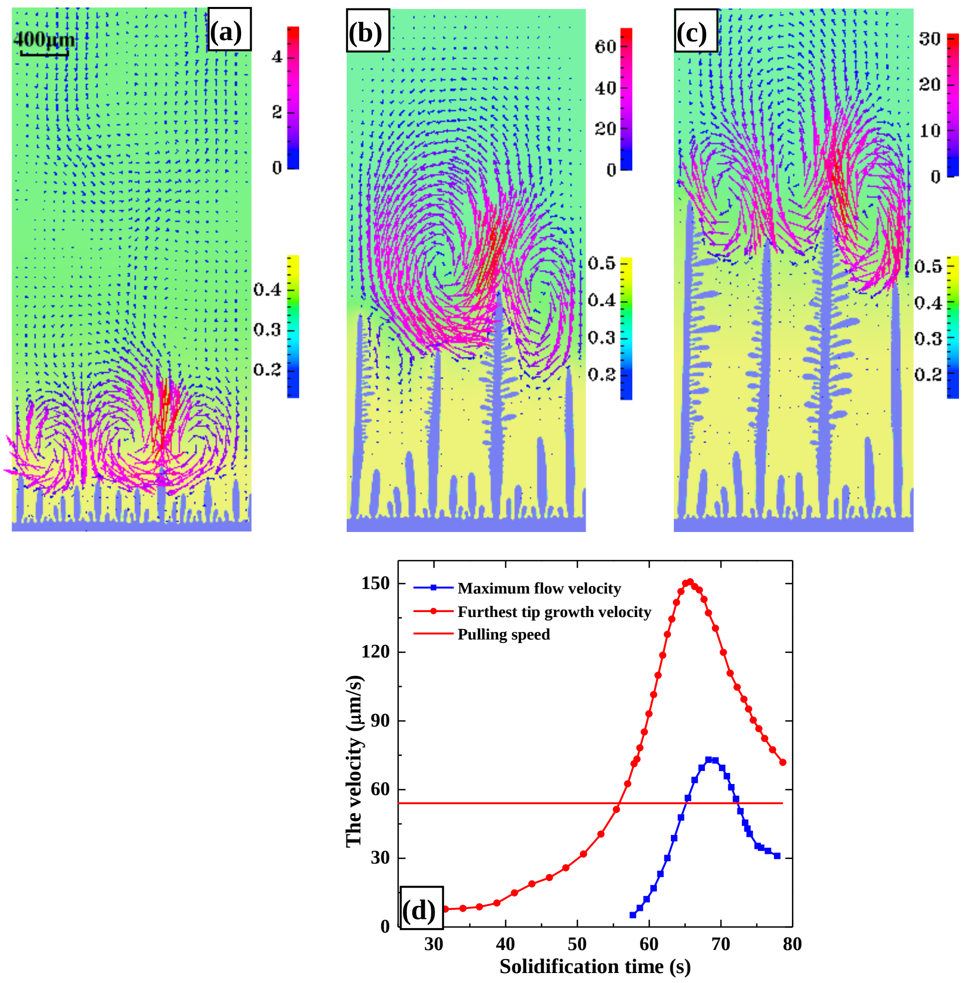

Supplementary Figure 21 | Time-dependent evolution of the phase-field simulated inter-dendritic flow of Fe-0.36 wt.% C steel. (a), (b) and (c) show the simulated liquid flow pattern, flow intensity ahead of the furthest solidification front and solute profile during solidification from the initial transient to the vicinity of steady state growth. The cooling rate R is 0.2 °C s^{-1} and temperature gradient G is 37 °C cm^{-1}. The upper legend shows the flow velocity (μm s^{-1}) and the lower legend shows the carbon concentration (wt.% C). (a) t = 57.75 s. (b) t = 70.19 s. (c) t = 77.85 s. (d) The variation of flow velocity and the growth velocity of the furthest columnar dendrite tip are plotted as a function of time.

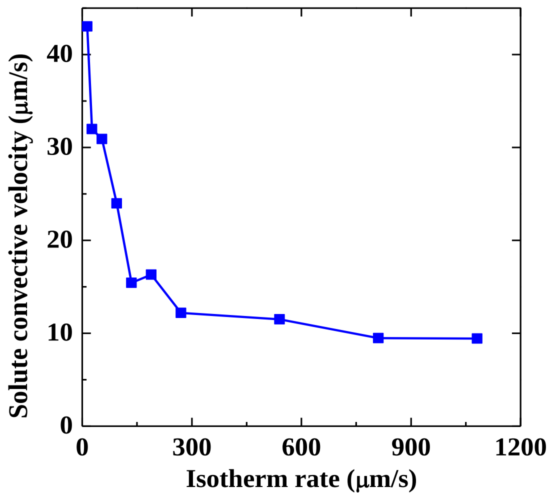

Supplementary Figure 22 | Simulated solute convective velocities with varying the isotherm rate. The solute convective velocity is obtained from the maximum flow velocity ahead of the columnar dendritic solidification front at nearly steady state growth in directional solidification of Fe-0.36wt.% C steel. The thermal gradient is fixed at G = 37 °C cm^{-1}.

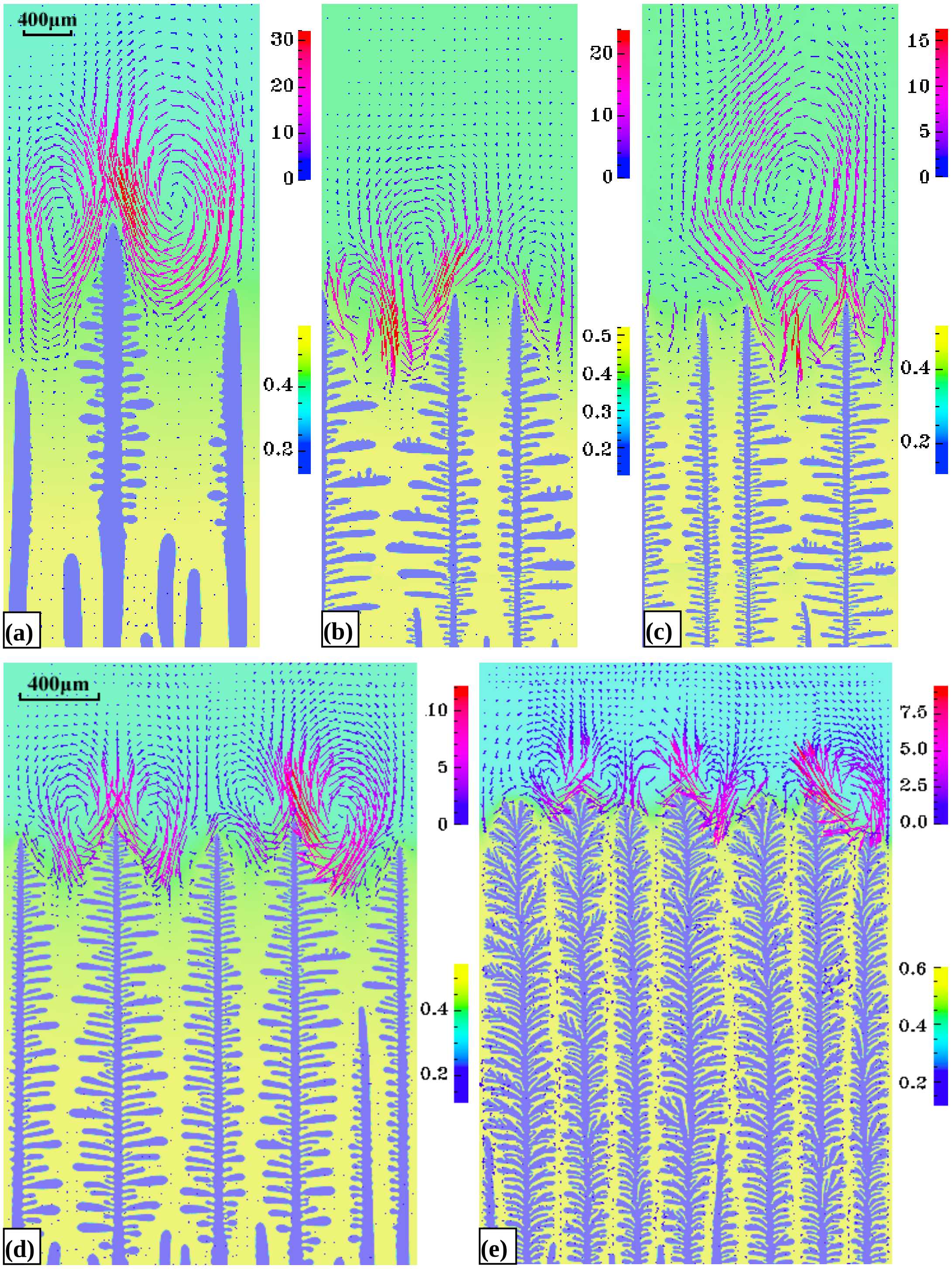

Supplementary Figure 23 | The flow fields and the solute profiles of Fe–0.36 wt.% C steel during directional solidification cooled at different rates. The simulations are at nearly steady state dendrite growth. The upper legend shows the flow velocity (μm s^{-1}) and the lower legend shows the carbon concentration (wt.% C). (a) R = 0.1 °C s^{-1}. (b) R = 0.35 °C s^{-1}. (c) R = 0.7 °C s^{-1}. (d) R = 1. 0 °C s^{-1}. (e) R = 3. 0 °C s^{-1}.

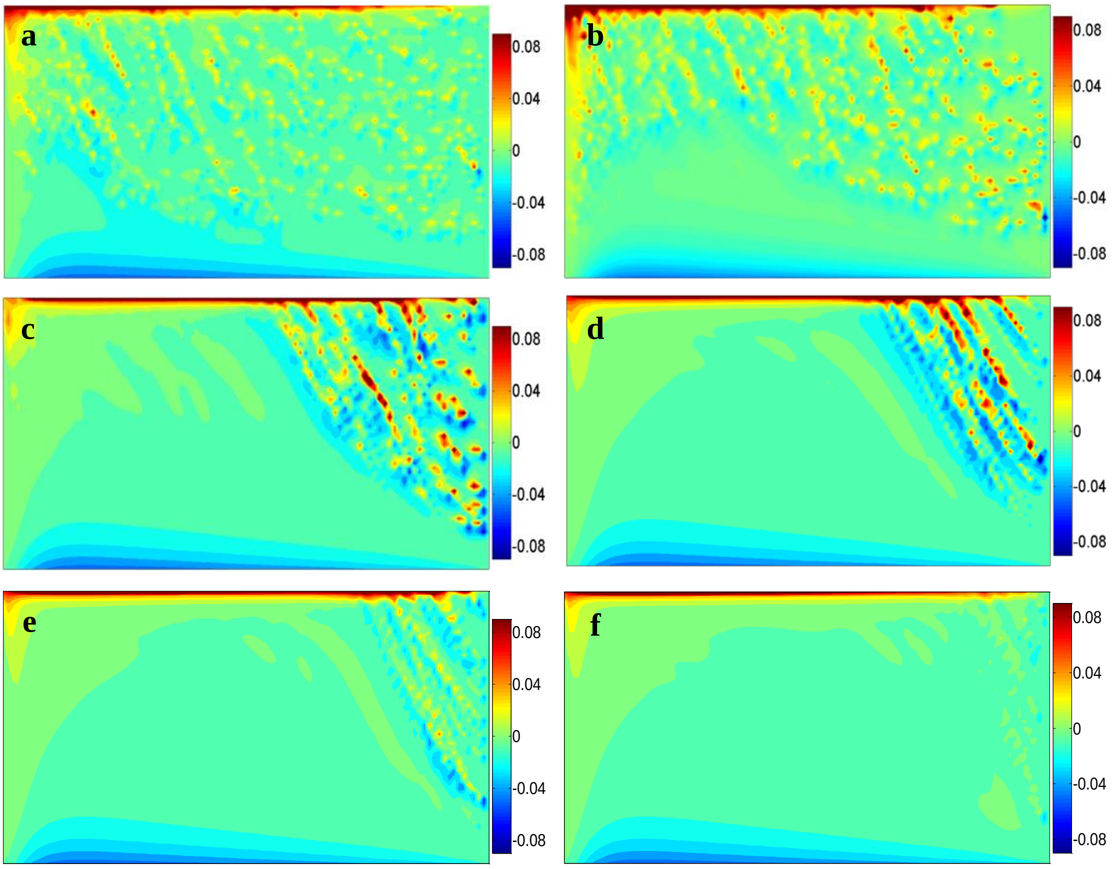

Supplementary Figure 24 | The effect of the dimension of particle on the CS formation of Fe-0.36 wt.% C steel in a unidirectionally solidified cavity (100 mm × 60 mm). The diameters of particles in a, b, c, d, e and f are 2, 5, 10, 15, 20 and 30 μm, respectively. The right legend denotes the relative segregation of carbon via the ratio of $(C\text{-}C_0)/C_0$.

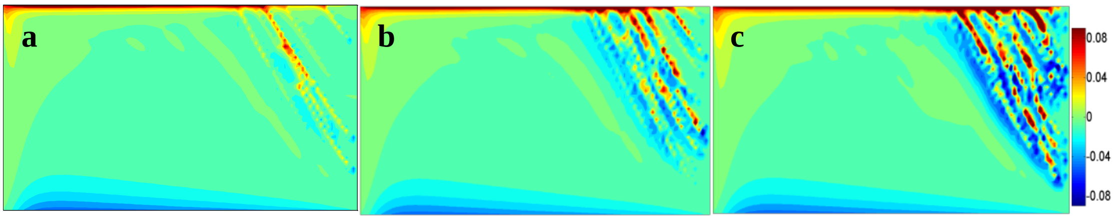

Supplementary Figure 25 | The effect of the particle quantity on the CS formation. For all three simulations the particle diameter is set to a value of 15 μm. The initial number of particles in a, b and c are 100, 500 and 1000, respectively.

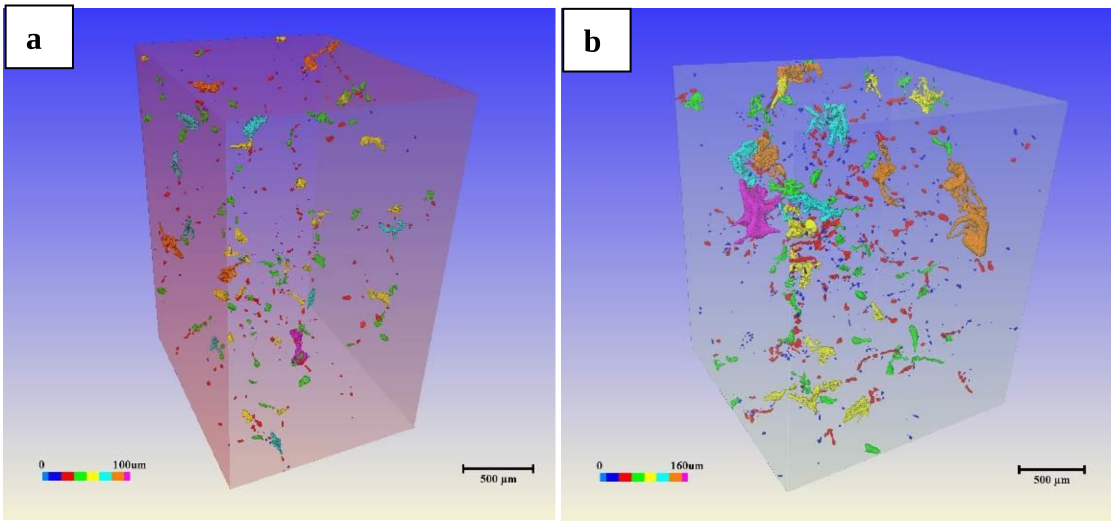

Supplementary Figure 26 | The morphology, dimensions and distributions of OIs detected by 3D HRTXRT. (a) At the adjacent onset site and (b) within the body part (b) of the CS in 1045 steel of **Experiment I**. The reconstructed dimensions of the left and right panels are 1800 μm × 1460 μm × 2500 μm and 1750 μm × 1830 μm × 2260 μm, respectively.

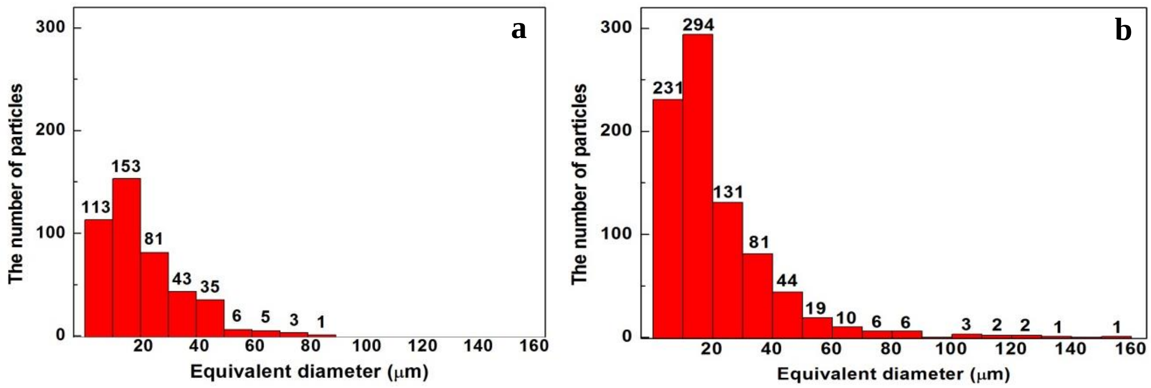

Supplementary Figure 27 | The statistics of the dimensions of OIs. (a) At the adjacent onset site and (b) within the body part of the CS in 1045 steel of **Experiment I.**

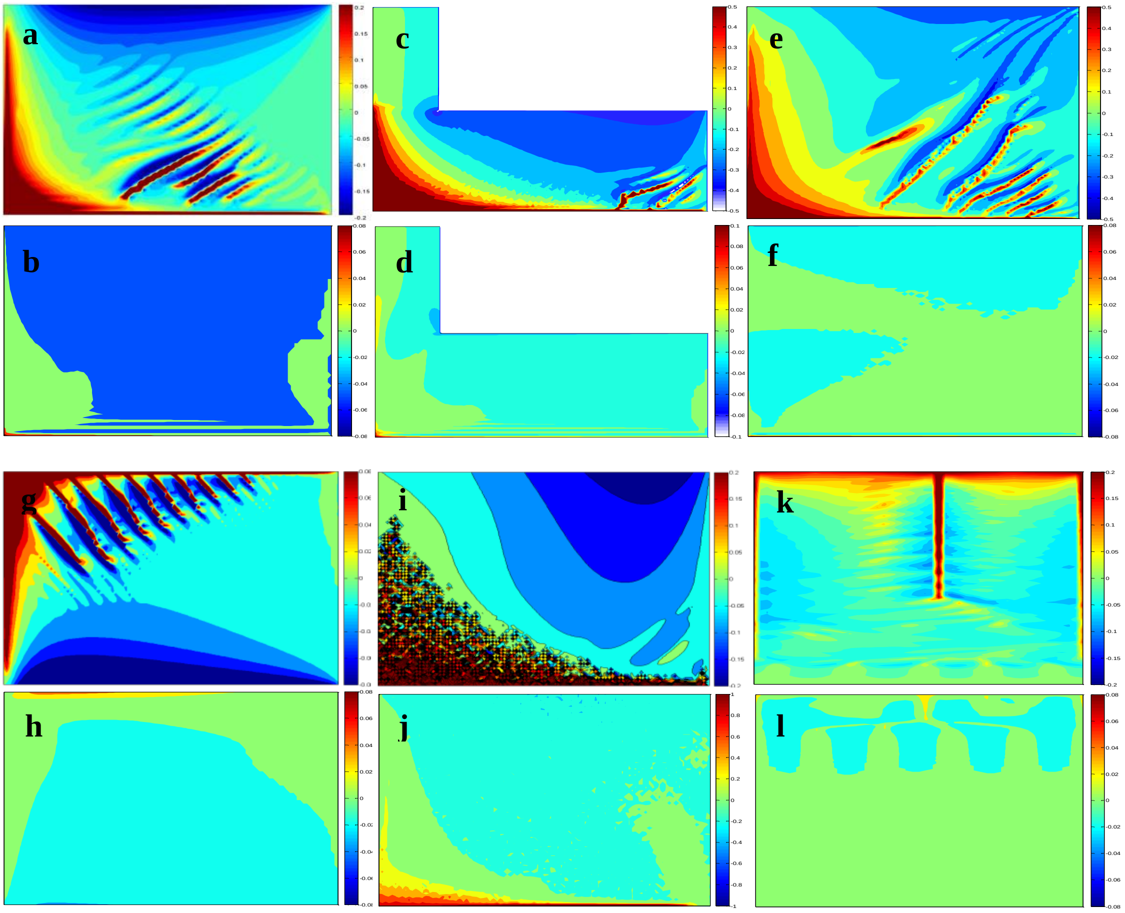

Supplementary Figure 28 | Simulated results of the CS in various systems. (a) and (b) are the Pb distribution maps of Sn-Pb alloy with the Pb concentrations (wt.%, the same below) of 5 and 0.36, respectively. (c) and (d) are the Cu distribution maps of Al-Cu alloy with the Cu concentrations of 20 and 0.2, respectively. (e) and (f) are the Bi distribution maps of Sn-Bi alloy with the Bi concentrations of 20 and 0.36, respectively. (g) and (h) are the C distribution maps of Fe-C system with solute expansion coefficient of 0.14 and 0.014 $(\text{wt. }\%)^{-1}$, respectively. (i) and (j) are the In distribution maps of Ga-In alloy with In concentrations of 5 and 1, respectively. (k) and (l) are the Ti distribution maps of Ni-based superalloy with Al, W, Ti concentrations of 5.6, 13, 1 and 0.56, 1.3, 0.1, respectively.

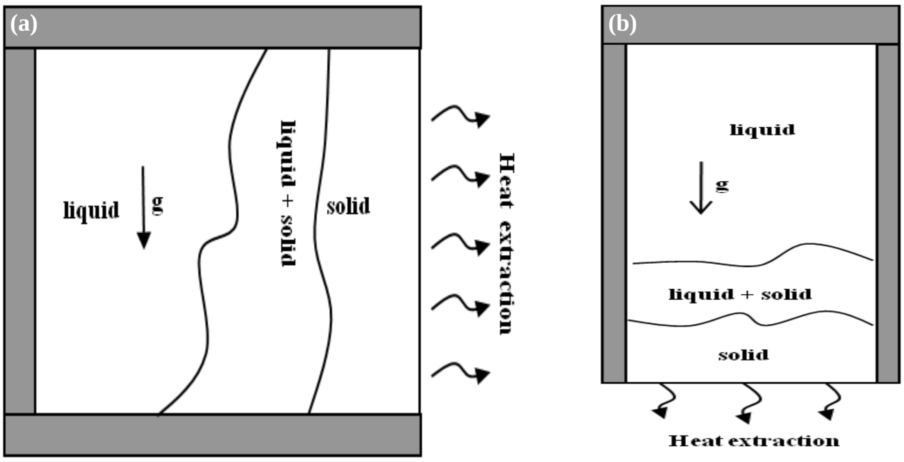

Supplementary Figure 29 | Schematic illustrations of two types of solidification. The cavities are cooled down from the right-hand side (a) and from the bottom (b).

Supplementary Table 1 | Operating conditions and obtained results of eighteen designed experimental ingots weighted from 0.5 to 650 tons. The AD and VCD techniques denote the aluminium deoxidation and the vacuum carbon deoxidation, respectively. T.O, C, S and P represent the average total oxygen, carbon, sulfur and phosphorus concentrations, respectively.

Expt.	Condition				Result				
	Composition	Mass (ton)	Poured method	Deoxi-dation	T.O (10^{-3} wt.%)	C (wt.%)	S (wt.%)	P (wt.%)	CS
**I**	45C	0.5	Air	AD	5.6	0.47	0.016	0.020	Serious
**II**	45C	0.5	Vac	VCD	1.0	0.47	0.005	0.005	No
**III**	45C	0.5	Vac	VCD	1.5	0.44	0.013	0.006	Very slight
**IV**	07Cr10W2V	0.5	Vac	VCD	2.0	0.07	0.005	0.007	Slight
**V**	45C	0.5	Air	VCD	0.7	0.45	0.008	0.009	No
**VI**	30Cr2Ni4MoV	100	Vac	VCD	1.0	0.22	0.005	0.006	No
**VII**	30Cr2Ni4MoV	100	Vac	AD	1.5	0.22	0.002	0.005	Slight
**VIII**	30Cr2Ni4MoV	100	Vac	VCD	1.2	0.22	0.003	0.005	No
**IX**	45C	5.0	Air	AD	3.6	0.49	0.018	0.026	Serious
**X**	45CrMoV	5.8	Air	AD	0.7	0.45	0.003	0.010	Very slight
**XI**	42CrMo	14	Air	AD	1.0	0.41	0.003	0.009	No
**XII**	42CrMo	16	Air	AD	0.8	0.42	0.002	0.015	Slight
**XIII**	42CrMo	16	Air	AD	1.1	0.45	0.002	0.008	Slight
**XIV**	34CrNiMo6	20	Air	AD	1.0	0.35	0.004	0.005	Very slight
**XV**	12Cr2Mo1	69	Vac	VCD	1.5	0.15	0.005	0.010	No
**XVI**	2.25Cr1Mo0.25V	234	Vac	VCD	1.2	0.14	0.004	0.007	No
**XVII**	30Cr2Ni4MoV	535	Vac	VCD	1.3	0.22	0.002	0.003	No
**XVIII**[3]	3.5NiCrMoV	650	Vac	VCD	**Good cleanliness	0.23	0.001	0.003	Very slight

**Good cleanliness denotes the little non-metallic inclusions in the 650 ton 3.5NiCrMoV steel ingot[3] treated via the VCD technique.

Supplementary Table 2 | The chemical compositions analysis of the CS and the adjacent zones (wt.%).

Elements	C	Si	Mn	P	S	Fe
Nominal composition	0.47	0.26	0.54	0.020	0.016	balanced
Sample no.1	0.53	0.32	0.58	0.020	0.020	balanced
Sample no.2	0.51	0.30	0.55	0.015	0.019	balanced
Sample no.3	0.48	0.30	0.56	0.018	0.014	balanced

Supplementary Table 3 | Main parameters and density contrasts calculated in various systems[4-11]. The parameters of *m* and *k* are the liquidus slope through the equilibrium phase diagram and the solute partition coefficient, respectively. β_T and β_C are the thermal and solutal expansion coefficients, respectively. $\Delta\rho/\rho_0$ (ρ_0 refers to the density of the bulk liquid and $\Delta\rho$ is the density difference between the mushy zone with a solid fraction of 0.3 and bulk liquid) denotes the density contrast of a given system.

Alloy system	*m*			*k*			β_C (10^{-2})			β_T (10^{-4})	$\frac{\Delta\rho}{\rho_0}$ (10^{-3})
Fe-0.45C-	C	Si	Mn	C	Si	Mn	C	Si	Mn		
0.3Si-0.8Mn[a]	-78	-17.1	-3.32	0.34	0.59	0.75	1.1	1.19	0.192	1.07	0.89
Fe-0.27C-	C	Si	Mn	C	Si	Mn	C	Si	Mn		
1.1Si-1.2Mn[b]	-78	-17.1	-3.32	0.34	0.59	0.75	1.1	1.19	0.192	1.07	2.1
Sn-5Pb		-1.286			0.0656			-0.53		0.60	-10.6
Sn-20Bi		-1.35			0.28			-0.30		1.00	-18.4
Al-4.4Cu		-3.39			0.171			-0.87		0.495	-13.4
Ga-5In		-0.677			0			-0.166		1.19	-3.7
Ni-5.6Al-	Al	W	Ti	Al	W	Ti	Al	W	Ti		
13W-1Ti[c]	-15.4	-0.64	-16.3	0.913	1.32	0.49	2.5	-0.5	0.82	1.40	12.3

[a]1045 steel in ASTM;

[b]27SiMn steel according to the classification definition in China;

[c] It needs to be noted that for a multi-component system (i.e., 1045 steel, 27SiMn steel and Ni-based superalloy) the density contrasts are derived through a sum of the density difference of individual component but neglecting their interactions.

Supplementary Table 4 | Parameters used in the numerical simulations with natural convection on Fe–0.36 wt.% C alloy

Dynamic viscosity, μ	4.2×10^{-3} Pa·s
Density of liquid steel, ρ_l	6.99 g cm^{-3}
Gibbs-Thomson coefficient, Γ	1.9×10^{-5} cm·°C
The solute partition coefficient, k	0.314
The liquidus slope, m	−80.45 °C $(wt.\%)^{-1}$
The anisotropy strength of surface tension, ε_4	0.030
The solute diffusion coefficient in liquid, D_l	2.0×10^{-4} cm^2 s^{-1}
The solute diffusion coefficient in solid, D_s	5.817×10^{-7} cm^2 s^{-1}
The initial solute concentration, C_0	0.36 wt.%
Reference temperature, T^*	1511 °C
Reference solute concentration, C^*	0.36 wt.%
Thermal expansion coefficient, β_T	-1.07×10^{-4} $°C^{-1}$
Solute expansion coefficient, β_C	-1.4164×10^{-2} $wt.\%^{-1}$
Gravity acceleration, g	−9.8 m^2 s^{-1}
Cooling rate, R	0.05 ~ 4.0 °C s^{-1}
Vertical temperature gradient, G	37 °C cm^{-1}
Interface width parameter, ξ	108

SUPPLEMENTARY NOTE 1: Simulated solidification process of 100-ton ingot

In the practical industrial process, the speed of solidification heavily depends on the mass of ingots. The larger the mass of ingot is, the longer the solidification time is. Relying on the classical macro-segregation theory[12], the heavy ingot will unavoidably result in the occurrence of severe CS. In order to estimate the solidification time and speed for huge ingots, as an example here we have simulated a 100-ton 30Cr2Ni4MoV ingot.

As illustrated in Supplementary Figure 1, the dimension of the ingot has a diameter of 2360 mm with a body height of 2370 mm. The Procast software[13] has been employed to simulate the 100-ton ingot with the proper options: (1) the node number of the discretized mesh was 192103 and the element number was 977389; (2) the material parameters for 30Cr2Ni4MoV steel ingot have been directly selected by Procast and the mould was made of the grey cast iron; (3) the boundary conditions have been selected with a heat transfer coefficient of 10 W $(m^2 \cdot K)^{-1}$ between mould and air at the room temperature (20 °C); (4) the initial temperature has been defined to be 1565 °C for ingot, 80 °C for the mould, 800 °C for insulations and 20 °C for the environment and covering flux. Our results demonstrate that the solidification time of its 100-ton ingot body will last 25 hours and the whole of time for the full solidification lasts over 36 hours. The temperature field for the solidification of the ingot body is further visualized in Supplementary Figure 2. Therefore, the average cooling rate of the ingot is as low as about 3~5 °C h^{-1}. For more details, please refer to our recent publication[1].

SUPPLEMENTARY NOTE 2: 2.25Cr1Mo0.25V 234-ton top poured ingot[14]

Experiment **XVI** (Supplementary Table 1) of a 234-ton 2.25CrlMo0.25V steel ingot with C 0.144, Si 0.08, Mn 0.56, S 0.004, P 0.007, Cr 2.40, Mo 1.03, V 0.27, T.O 0.0012 and Fe balanced in its chemical compositions (wt.%) was produced by the electric arc furnace-ladle furnace-vacuum degassing-mould stream degassing and the VCD technique was adopted. The solidification structure, macrosegregation and porosity of the ingot were checked by means of acid dipping experiment, and the results showed no CS in the ingot body.

SUPPLEMENTARY NOTE 3: 30Cr2Ni4MoV 535-ton top poured ingot

Experiment **XVII** **(**Supplementary Table 1) of a 535-ton 30Cr2Ni4MoV steel ingot with C 0.22, Si 0.02, Mn 0.30, S 0.002, P 0.003, Cr 1.71, Ni 3.55, Mo 0.38, V 0.10, T.O 0.0013 and Fe balanced in its chemical compositions (wt.%) was produced by the electric arc furnace-ladle furnace-vacuum degassing-mould stream degassing and the VCD technique was adopted. This ingot has its maximum diameter of 4100 mm. The macrosegregation and porosity of the ingot were checked by means of acid dipping experiment, and the results showed no CS in ingot body. This ingot was fabricated by our collaboration partner in the heavy metal industries and the current results are unpublished.

SUPPLEMENTARY NOTE 4: Experimental observations of the oxide-based inclusions in CS

1) Experimental procedure

The CS samples were cut from the steel ingots in **Experiment I** and **VII** for the inclusions and cavities analysis. After the samples were grinded, polished and etched by the 3wt.%HNO_3-C_2H_5OH solution, the inclusions and cavities in the CS were analyzed and identified by the scanning electron microscopy (SEM) and energy dispersive spectra (EDS).

2) The alumina in the CS

Supplementary Figure 10 illustrates the morphology and the distribution of alumina in the CS zones. It can be seen that the alumina is generally cluster-like and the alumina among the cluster is polyhedral (see Supplementary Figure 10a). Occasionally, there exists the separated granular alumina in Supplementary Figure 10b.

3) The MnS in the CS

Supplementary Figures 11 and 12 show the MnS inclusions observed in CS zones. The MnS inclusions can be categorized into two types, according to their sizes and morphologies. The first type of MnS usually exceeds 100 μm with the strip-like shape, shown in Supplementary Figure 11a. Some MnS clusters with a skeletal morphology also look like a strip in the whole (see Supplementary Figure 11b). The second type of MnS usually has the sizes of about 2~5 μm featured by the dendritic, acetabular or granular morphologies as evidenced in Supplementary Figures 12a-12d.

4) The coexistence of alumina and MnS/S element

Interestingly, in the CS the coexistence of alumina and MnS is regularly observed. Supplementary Figures 13 and 14 show the morphologies and chemical compositions of the cluster-like alumina and MnS. Supplementary Figures 13b and 14a visualize clearly their shapes and distribution in the microscale. The EDS analysis evidences that the coexistence of alumina and MnS regions as illustrated in Supplementary Figures 13c-13d and 14b-14c, respectively. In some observed regions, although there

is no coexistence of MnS with alumina (see Supplementary Figure 15a) the S element enrichment near the alumina has been confirmed by the element distribution mapping produced by EDS, as shown in Supplementary Figures 15b-15d.

5) The cavities in CS

Some bubble-like cavities have been found in the CS, shown in Supplementary Figure 16. The inner wall of the cavities is rather smooth without dendritic feature, which means that gas should exist in the formation process of cavities.

6) Chemical analysis of main elements distributions in the CS

A chemical analysis of elements distribution in the region of the CS in **Experiment I** has been performed to give a clear understanding of the main elements distributions in the CS. A cuboid sample with a size of 40 mm × 150 mm × 20 mm covering the region of the CS has been first cut from the etched longitudinal sections. Three zones (named samples no.1, no.2, and no.3) with a diameter of about 5 mm are then selected to perform spectrum analysis. The samples no.1 and no.2 are within the region of CS and the sample no.3 is near their locations (near the region of CS). The chemical compositional analysis is further compiled in Supplementary Table 2. In comparison with the nominal composition of the steel and the chemical composition of sample no.3, the elements C, S, Si and Mn have been found to segregate positively in the CS zones, whereas the segregation of P has not been obvious.

7) Experimental characterisations of the quantities and sizes of OIs in CS

In order to elucidate the volume fraction of OIs in CS in the steel ingot (**Experiment I**), the 3D high resolution transmission X-ray tomography (HRTXRT) technique has been employed with the lab-based Xradia Versa XRM-500 system. X-ray tomography imaging was performed on the smaples with a cylindrical (Φ3 mm × 25 mm) or rectangular (4 mm × 4 mm × 35 mm) shapes cut from the CS. The working accelerating voltage was 140 kV. In total, for each sample 1600 images, each of which has an exposure time of 4 seconds, were recorded as the sample was rotated by 360°, and were then reconstructed computationally to produce a 3D image through a filtered back projection algorithm with a voxel size of about 3.5 μm. Compared with the destructive metallographic techniques, the 3D HRTXRT allows a nondestructive

visualization of the inner structures and some quantitative informations, such as the size, volume, distribution and 3D connectivity of OIs in the CS. In addition, the HRTXRT results are reliable, because there are no artificial defects induced from the traditional grinding and polishing processes during the preparation of samples[15].

Supplementary Figure 26 shows the morphology, dimensions and distributions of OIs at the adjacent onset site and within the body of the CS. The results demonstrate that both volume fraction and the size of the OIs within the body of the CS are larger than those at the adjacent onset site. This is mainly because, after the nucleation of the CS, the OIs coagulate and grow into large sizes during the development of channels. The adjacent OIs can find their ways to preferentially float into the channels together, finally leading to the increase of quantity and dimension for the OIs after solidification. The measured average volume fractions of OIs at the adjacent onset site and within the body parts of the CS are 0.09% and 0.35%, respectively. In particular, it needs to be emphasized that those OIs with dimensions being smaller than the spatial resolution (3.5 μm) can not be detected by our current X-ray tomography technique.

Furthermore, the statistical results for those OIs measured here has been compiled in Supplementary Figure 27. It can be seen that the diameters of OIs are mainly in the range of 5~50 μm. In the sample within the body of the CS, the total number and size of OIs increase significantly, as compared with those at the adjacent onset site of CS. In particular, within the body of the CS, we have observed some large OIs with a diameter above 100 μm, which indeed was not detected at the adjacent onset site.

SUPPLEMENTARY NOTE 5: First-principles calculations of inclusion formation

1) Models

The simulation mainly focuses on the interactions between S atom, Mn + *n*S atom complex and the surface of solid alumina in the steel melt. To establish the model system, we have first made two assumptions. They are (1) that S element exists as S atom and Mn + *n*S atom complex in the melt, and (2) the interfacial interaction of alumina, S atom and Mn + *n*S atom complex with the iron melt is ignored.

The crystal structure of α-alumina (α-Al_2O_3) with the conventional hexagonal cell is shown in Supplementary Figure 17a. The optimized lattice constants, enthalpy of formation [ΔH = −153.99 kJ (mole of atoms)$^{-1}$], bulk band gap (E_g = 5.9 eV at the Γ-point) of bulk α-Al_2O_3 are in agreement with the previously reported results[2,16,17]. Along the [0001] direction, the stacking sequence can be viewed as … O-Al-Al-O-Al-Al-O …. The distance between any two adjacent O layers is about 2.2 Å in its bulk equilibrium phase. Thus, three (0001) surface terminations[2,16,17] are available, namely, Al-, AlO- and O-terminated surfaces (see Supplementary Figure 17b). Consistent with the reported results[2,16], the clean AlO-terminated (0001) surface is energetically most favorable, with the lowest surface energy of 1.68 J m^{-2}. Therefore, all our calculations are based on the non-dipole AlO-terminated surface, which denotes a single outmost Al-layer plus a sub-outmost O-layer. The optimized surface structure is illustrated in Supplementary Figure 17c, indicating that the space distance between the topmost layer and the second layer is significantly reduced ($\Delta l \approx$ 0.738 Å being about 78% of the unrelaxed distance as shown in Supplementary Figure 17b). These results show that the topmost Al atom is now almost in the same layer with the second oxygen layer. This situation is mainly due to that the coordination number of the topmost Al atom is much less, as compared in the crystal. In order to simulate the adsorption on the surface of α-Al_2O_3, we have built the 18-layer-thickness slab with a 15 Å vacuum depth along the [0001] direction, together with the 2 × 2 dimension surface unit cell. For all surface calculations, the bottom

nine layers are always kept frozen and the other nine layers are allowed to be relaxed. The adsorption energy E_{ads} for S atoms and Mn + nS atom complex on alumina surface, which is proposed to evaluate the strength of adsorption, can be derived as Eqs. (1) and (2), respectively.

$$E_{ads} = E(\mathrm{S} + \mathrm{alumina}) - E(\mathrm{S}) - E(\mathrm{alumina}) \quad (1)$$

$$E_{ads} = E(\mathrm{MnS}_n + \mathrm{alumina}) - E(\mathrm{MnS}_n) - E(\mathrm{alumina}) \quad (2)$$

where E (S + alumina) is the energy of surface with the S atom adsorption, E (MnS_n + alumina) is the energy of surface with the Mn + nS atom complex adsorption, E (S) is the energy of the free S atom, and E (MnS_n) is the energy of the Mn + nS atom complex and E(alumina) is the energy of clean surface.

And the bonding energy E_{bind} for single S atom with Mn + nS atom complex is calculated as Eq. (3).

$$E_{bind} = E(\mathrm{MnS}_{\mathrm{n+1}} + \mathrm{alumina}) - E(\mathrm{MnS}_{\mathrm{n}} + \mathrm{alumina}) - E(\mathrm{S}) \quad (3)$$

where E (MnS_{n+1} + alumina) and E (MnS_n + alumina) are the total energies of the MnS_{n+1} and MnS_n atom complexes adsorbed on the surface, respectively.

2) Adsorption of S atom on the surface of α-Al_2O_3

Based on the S enrichment near the alumina observed in experiment, we simulate the adsorption of S atom on the surface of α-Al_2O_3. The five possible adsorption positions on the AlO-terminated surface are shown in Supplementary Figure 17d, which are Al1, Al2 and Al3 on the top of aluminium atoms in the topmost, third and fourth atoms layers and O1 and O2 on the top of oxygen atoms in the second and fifth atomic layers. The surface relaxation calculations reveal that S has two stable adsorption sites on the alumina surface. The initial adsorption sites on O1, Al2 and Al3 finally converge into the same local environment site with the most stable adsorption energy of about -2.0 eV. In this situation, the trapped S atom is tightly bounded to two nearest neighboring Al and O atoms through the S-Al’ bonding length of 2.211 Å and the S-O’ bonding length of 1.828 Å, as illustrated in Supplementary Figure 18a. While, the initial S adsorptions on O2 and Al1 sites are both relaxed into an identical site with a much less adsorption energy of about −0.82 eV. In this case,

the trapped S atom just binds with its nearest neighboring Al atom through the S-Al' bonding length of 2.253 Å, as illustrated in Supplementary Figure 18b.

In order to further elucidate the adsorption mechanism of the S atom on the surface of α-Al_2O_3, we have analyzed the electronic structures, shown in Supplementary Figure 19. The obtained electronic band structure for the constructed slab evidences that the S adsorption on AlO-terminated surface is an insulator with the direct band gap of 2.1 eV at the Γ point (see Supplementary Figure 19a). The top band consists of a highly pure S-p-like state whereas the bottom of the conduction band is composed of the empty S-d-like states with a small part of S-p-like states. Importantly, in the bonding region of the energy region from −2.0 eV to −1.2 eV, there is a clear hybridization between the p-like states from the trapped S atom and from its nearest neighboring oxygen (O') atom on the surface (see Supplementary Figure 19b), evidencing a S-O' bonding interaction. On the other hand, in the low-lying energy region from −6.3 eV to −8.0 eV, the s-like state of the trapped S atom exhibits a very similar profile to the s-like state of its nearest neighboring Al atom (Al') (see Supplementary Figure 19c). These similar DOS shapes uncover that the bonding interaction indeed exists between the trapped S atom and its nearest neighboring Al atom. Therefore, the S adsorption on the AlO-terminated surface can be ascribed to these two kinds of bonding interactions between S and its nearest oxygen atom, and between S and its nearest Al atom. In order to further identify the bonding character, we have calculated the charge accumulations and depletions for all these atoms of the surface using Bader's technique[18,19]. For the sake of comparison, the charges of the clean AlO-terminated surface have been calculated. In terms of our PAW calculations within the denser 200 × 200 × 300 k mesh (12 million grid points), it has been found that the Al and oxygen atoms on the clean surface have the charges of 0.55e and 7.6e, respectively. However, after the S adsorption on the AlO-terminated surface, the charge accumulations and depletions are significantly changed for surface atoms surrounding the S adsorption site: (1) its nearest neighboring oxygen atom (see O' in Supplementary Figure 18a) now has a charge of 7.3e, which is less by 0.3e than that (7.6e) before the occurrence of the S adsorption, (2) its nearest neighboring Al' atom

now has only 0.53e, almost unchanged before and after the occurrence of the S adsorption, and (3) the S atom itself has a charge of 6.29e which implies that S obtains the charge of about 0.29e. In comparison with the charges of the clean surface as discussed above, it can be deduced that the S adsorption leads the charge of its nearest neighboring Al' atoms to be transferred not only to O' (1.3e) but also to the trapped S atom (0.29e). Therefore, the S-Al' bonding feature is ionic. Although there is no occurrence of the charge transfer between S and its nearest neighboring oxygen (O'), the existence of the strong hybridization of their p-like states between the S and O' atoms (see Supplementary Figure 19b) evidences that their S-O' bond is characteristic of the covalent hybridization. Besides that, the charge of the other oxygen and Al atoms on the surface remains almost unchanged before and after the S adsorption.

3) Adsorption of Mn + *n*S atom complex on the surface of α-Al_2O_3

Based on the coexistence of MnS and alumina observed in experiment, we have simulated the adsorption of Mn + *n*S atom complex on the surface of α-Al_2O_3. Firstly, five possible adsorption positions (O1, O2, Al1, Al2 and Al3) are investigated for Mn atom on the AlO-terminated surface. The simulated results show that the lowest energy, equilibrium, adsorption sites of Mn on the AlO-terminated surface is found to be a centre of the triangle formed by three O1 atoms on the second layer from top and is located above Al3 atoms in the fourth layer from top. The adsorption energy for Mn atom calculated by Eq. (2) is −1.97 eV. The Mn bond distance to the nearest neighbor oxygen (O1 atom) and aluminium (Al3) atom are 2.17 Å and 2.88 Å, respectively, with an O1-Mn-O1 angle of 73.6° and the Mn atom is located 1.673 Å above the cell surface.

Based on the adsorbed Mn, we have further calculated the formation of Mn + *n*S atom complex on the AlO-terminated surface according to Eq. (2). Figure 3b in the main text compiles the adsorption energies of Mn + *n*S atom complex as a function of the number (*n*) of the S addition, revealing the occurrence of the stable adsorption of Mn + 1S and Mn + 2S atom complex with the adsorption energies of −0.71 eV and −0.23 eV, respectively. The adsorption geometries are further illustrated in

Supplementary Figure 18. It has been seen that, with the presence of S atom, the adsorbed Mn atom is bounded with the two nearest O1 atoms with the Mn-O1 length of 2.15 Å in the Mn + 1S case and 2.10 Å in the Mn + 2S case, respectively. These Mn-O1 bonds are significantly shorter than that (2.17 Å) in the case only with the Mn atom adsorption. It has been also noted that the trapped S atoms are bounded with the Al1 atom on the surface with the same bonding length of 2.26 Å for both the Mn + 1S and Mn + 2S cases. Besides that, the S atom is also strongly bounded with Mn through the distance of 2.24 Å in the Mn + 1S case and of 2.22 Å in the Mn + 2S case. Another S atom in the Mn + 2S case is only strongly bounded to Mn with the relatively short bond of 2.09 Å. However, once the third S atom is introduced to bind with Mn, the adsorption energy becomes unstable. The most noticeable change is the increasing of two corresponding Mn-O1 bond lengths in the Mn + 3S case compared with Mn + 1S and Mn + 2S cases, reflecting well the weakening of the adsorption energy. These bond lengths get larger in the Mn + 4S case as accompanied with the nearly zero adsorption energy, which suggests the detachment of Mn + 4S atom complex from the surface of α-Al_2O_3. Furthermore, we have calculated the binding energy for the extra-introduced S with the already formed Mn + *n*S atom complex according to Eq. (3). It has been revealed that the strong attractive interaction between S and Mn + *n*S atom complex exists before and after the detachment of Mn + *n*S atom complex from the surface (see Figure 3c in the main text).

Interestingly, calculation implies that the surface of alumina could adsorb Mn. As shown in Supplementary Figure 20, the calculated spin-polarized densities of states for the Mn on the surface reveal a strong electronic hybridization between Mn d-like state and its three nearest neighboring oxygen (O1) p-like states in the energy region from −10 eV to −3 eV below the Fermi level. Furthermore, Mn is also found to carry a highly large local spin magnetic moment of about 4.0 μ_B, suggesting that the magnetism of Mn actually also plays a role in stabilizing the Mn adsorption. In addition, we note that there are two sharp peaks at −1.0 eV and −1.4 eV appearing in the energy region from −3.0 eV to the Fermi level, which can be attributed to highly pure Mn d-like state. Specifically, the spin-up peak at −1.0 eV is mainly composed of

Mn $d_{(x^2-y^2)}$ and d_{xy}-like states whereas the spin-down peak at −1.4 eV consists of d_{xz} and d_{yz}-like states.

Further, our theoretical simulation results demonstrate that the Mn + 1S and Mn + 2S atom complexes can be adsorbed successfully. However, the adsorption energies are significantly reduced to −0.7 eV and −0.2 eV, respectively. This is made clear in Supplementary Figure 20, where the strong hybridization is shown between S and Mn in the energy region from −4 eV to the Fermi level. However, the hybridization between the O p-like densities from its nearest neighboring oxygen (O1) and Mn d-like states is significantly reduced with respect to the case only trapping Mn atom. This fact evidences the weakening of the Mn-O1 chemical bonding, depending on the number of S atoms binding with Mn. Therefore, the formation of larger Mn + *n*S atom complex on the surface drives itself to detach from the AlO-terminated surface. It helps to explain the experimental observation that MnS separates from the surface of Al_2O_3.

SUPPLEMENTARY NOTE 6: Mesoscale simulations of interdendritic melt convection

1) The phase-field model with incorporation of fluid flow dynamics

The computer simulations in mesoscale are mainly to understand the intensity of the thermosolutal convection among the formed dendrites of iron alloy, and therefore the quantitative phase-field model[20] in coupling of the fluid flow dynamics has been employed for calculations. In this corporation of models, the evolution of the solid-liquid interface is governed by the phase-field model, while the movement of the liquid phase is described by the incompressible Navier-Stokes equations. In the phase-field model, the order parameter ψ has a constant value in solid (ψ = 1) and liquid (ψ = − 1) phases, and varies smoothly across the thin diffuse interface. The governing equations[20,21] of the phase-field model in two dimensions are listed as follows,

$$\tau_0\left(1-(1-k)\frac{y-V_P t}{l_T}\right)a^2(\mathbf{n})\frac{\partial\psi}{\partial t}=\nabla\cdot\left(W^2(\mathbf{n})\nabla\psi\right)+\partial_x\left(|\nabla\psi|^2 W(\mathbf{n})\frac{\partial W(\mathbf{n})}{\partial(\partial_x\psi)}\right)$$
$$+\partial_y\left(|\nabla\psi|^2 W(\mathbf{n})\frac{\partial W(\mathbf{n})}{\partial(\partial_y\psi)}\right)+\left[\psi-\lambda I\left(1-\psi^2\right)\right]\left(1-\psi^2\right) \quad (4)$$

where x is the horizontal direction and y the vertical direction, τ_0 is the characteristic dissipation time, $W(\mathbf{n}) = W_0 a(\mathbf{n})$, $\mathbf{n} = \nabla\psi/|\nabla\psi|$, $a(\mathbf{n}) = W_0(1-3\varepsilon_4)[1 + 4\varepsilon_4 \frac{(\partial_x\psi)^4+(\partial_y\psi)^4}{(1-3\varepsilon_4)|\nabla\psi|^4}]$, $\lambda = a_1\xi$, $\xi = W_0/d_0$, $d_0 = \Gamma/[|m|C_0(1-k)]$ is the chemical capillary length, Γ the Gibbs-Thomson coefficient, C_0 the nominal concentration of the liquid. ε_4 is the fourfold anisotropy strength, $I = U + (y - V_P t)/l_T$, $l_T = |m|C_0(1-k)/G$ with G the temperature gradient. The pulling speed V_P in Eq. (4), which denotes the translation rate of isotherms in directional solidification by cooling down at rate R, is equal to R/G. The dimensionless solute concentration U is obtained by solving the solute conservation equation with the solute anti-trapping term introduced by Karma[20]. It is written including the terms describing the solute transport by convection

$$[(1+k)-(1-k)\psi]\frac{\partial U}{\partial t}+\mathbf{u}\cdot[((1+k)-(1-k)\psi)\nabla U-(1+(1-k)U)\nabla\psi]$$
$$=\nabla\cdot[Dq(\psi)\nabla U+\frac{1}{\sqrt{2}}(1+(1-k)U)\frac{\partial\psi}{\partial t}\frac{\nabla\psi}{|\nabla\psi|}]+[1+(1-k)U]\frac{\partial\psi}{\partial t} \quad (5)$$

where $D = a_2\lambda W_0^2/\tau_0$, $q(\psi) = [(1-\psi)+k(1+\psi)D_s/D_l]$, $C/C_0 = [1+(1-k)U][1+k-(1-k)\psi]/2$. D_l and D_s are the solute diffusion coefficients in liquid and solid, respectively. In this approach, the attachment kinetics at the interface is neglected so that a_1 = 0.8839, a_2 = 0.6267. Since in directional solidification, the temperature is controlled by the external thermal gradient and the growth of alloy crystal is controlled by the solute transports, therefore, the thermal diffusion equation is not solved together.

The melt is assumed incompressible and its motion is described by the Navier-Stokes equations:

$$\frac{(1-\psi)}{2}\nabla\cdot\mathbf{u}=0 \quad (6)$$

$$\rho_l[\frac{(1-\psi)}{2}\frac{\partial\mathbf{u}}{\partial t}+\frac{(1-\psi)}{2}(\mathbf{u}\cdot\nabla)\mathbf{u}]=\mu\nabla^2\mathbf{u}-\nabla p+\mathbf{S}\,. \quad (7)$$

where p is the pressure and the adding factor $(1-\psi)/2$ is to make sure that in the solid phase the liquid flow velocity is zero. The source term $\mathbf{S}$ in Eq. (7) which takes into account the gravity force induced by the liquid expansion is considered through the Boussinesq approximation[22].

$$\rho\mathbf{g}=\rho_l\mathbf{g}\left[1+\beta_T\left(T-T^*\right)+\beta_C\left(C-C^*\right)\right] \quad (8)$$

in which T^* and $C^*$are the reference values of temperature and solute concentration.

2) The implementation of numerical simulations

The numerical simulations of Fe–0.36wt.% C steel in upward directional solidification have been performed by solving the phase-field model using the finite element method. Heat extraction from the top boundary to the bottom is imposed by setting a constant vertical thermal gradient G. The solidification would take place by cooling both the hot and cold zones at a constant rate R. To enhance the computing efficiency, an adaptive mesh and parallel computing procedure[23,24] have been used in simulations. The physical properties of steel and computational parameters have been listed in Supplementary Table 4. To eliminate the influence of rejected solute on the

increasing of the concentration in the bulk liquid during solidification, a large rectangular simulation domain with a size of 2000 × 6500 (2117 μm × 6880 μm) has been chosen with the dimensionless minimum length of the adaptive mesh element size dx_{min} = 0.25. The dimensionless discretization time step was dt = 0.02 ~ 0.06. As it has been confirmed that in the previous study[24], a growing dendrite is dynamically interacting with the liquid flow and the historically influence of liquid flow on dendrite growth should be considered, hence the initial transient of the directional solidification has been taken into account in simulations (*i.e.*, the solidification occurred with a planar solid-liquid interface). Consequently, in the initial state the phase-field variable has been set as $\psi(\mathbf{x},0) = -tanh\ (y/\sqrt{2})$ along the normal to the planar interface, and the solute concentration has been initialized with $C_l = C_0$ in liquid and $C_s = kC_0$ in solid, which results in the value of dimensionless solute concentration U is zero in the whole domain. No flux Neumann conditions has been used for both ψ and C at outside boundaries of computation domain.

The momentum and mass conservation equations were solved using the modified projection method[25] by a semi-implicit algorithm. All the boundaries of the domain have been considered as the walls where the flow velocity is zero (*i.e.*, no-slip boundary condition has been adopted at all those walls). It should be noted that the vertical temperature gradient, considered as the top boundary, would be hotter than the bottom that is indeed in a stable configuration with respect to the density profile in the melt. As it would not provide a driving force for the hydrodynamic instability, the thermal convection can be neglected during the simulations. During the solidification of heavy steel ingot, it would be reasonable since the thermal convection between dendrites is negligible as compared with that of solutes[26].

3) The variation of the simulated thermosolutal convection with applied processing parameters

In the casting of ingots, the cooling rate and thermal gradient of solidifying melt are varying in a wide region, and thus in order to acquire a direct insight of thermosolutal convection in the inter-dendritic regions under different solidification

conditions, a variety of cooling rates (R = 0.05 ~ 4.0 °C s^{-1}) for a fixed temperature gradient G = 37 °C cm^{-1} have been taken into account in simulations. It has been long recognized that, at a given thermal gradient G, the morphology of the solid-liquid interface changes with the pulling speed or isotherm rate (R/G), so that the solute distribution ahead of the furthest solidification front will vary much with the changing of the cooling rate, in turn will result in different flow patterns and intensities among dendrites. Supplementary Figure 21 shows the time-dependent evolutions of solute distribution and melt flow ahead of the solidification front from the initial transient to the vicinity of the steady state growth at cooling rate R = 0.2 °C s^{-1}, and V_P = 54.05 µm s^{-1}. As the tip growth velocity of the furthest columnar dendrite reached near the isotherm rate, the maximum flow velocity before the furthest dendrite tip would not vary much with time as well. Hence, this maximum flow velocity has been considered as the characteristic velocity of the solutal flow in the inter-dendritic regions as shown in Supplementary Figure 22. For some of other simulated cases, the simulated flow, solute fields and the morphologies of dendrites at nearly steady state growth are illustrated in Supplementary Figure 23. In order to gain a clear understanding of the evolution dynamics of fluid flow and dendrite growth, the time evolution of the furthest tip velocity for R = 0.2 °C s^{-1}, is also imposed in Supplementary Figure 21d. These two curves demonstrate that the maximum flow velocity ahead of the solidification front is always below the furthest tip growth velocity, and therefore the rejected solute elements in the inter-dendritic regions have difficulty to move progressively to form segregated channels. This conforms with the results of all other simulated cases where the inter-dendritic solute convective velocity is weak (typically, ~10 µm s^{-1}), slower than the isotherm rate. Comparing to the strong solute convective velocity (100 µm s^{-1} ~ 1 mm s^{-1}) [27] in model alloys, the weak inter-dendritic melt convection strength in steel alloys is mainly ascribed to the much smaller density contrast between the solute-enriched melt and bulk liquid, which can be well approved in the following theoretical calculations and numerical simulations.

SUPPLEMENTARY NOTE 7: Density contrast calculations and process-scale simulations of CS in various systems

1) Density contrasts in various systems

In order to illustrate the different thermosolutal convection strength in various model alloy systems, such as Sn-Pb, Sn-Bi, Al-Cu, Ga-In, Ni-based superalloy, 1045 carbon steel and high Si steel, we have derived theoretically the density contrasts between inter-dendritic melt and bulk liquid. Within our current calculations, the volume fractions and compositions of the interdendritic liquid are approximated by the Scheil microsegregation model for substitutional elements (i.e., silicon in steel, and solutes in model alloys) and the equilibrium lever rule for interstitial elements (*e.g.*, carbon in steel). The local thermodynamic equilibrium at the solid-liquid interface is guaranteed by assuming that the temperature at the liquid side is equal to the liquidus temperature of the melt, as following

$$T = T_0 + mC_l \tag{9}$$

where C_l is the solute concentration at the liquid side of solid-liquid interface and T_0 is the melting point. Therefore, the density contrasts can be obtained in combination with the Boussinesq approximation[22],

$$\frac{\Delta\rho}{\rho_0} = C_0[(1-f_s)^{k-1} - 1](\beta_C + \beta_T m) \tag{10}$$

$$\frac{\Delta\rho}{\rho_0} = C_0 \frac{f_s(1-k)}{(1-f_s)(1-k)+k}(\beta_C + \beta_T m) \tag{11}$$

where f_s is the solid fraction. It should be noticed that Eq. (10) stems from the Scheil model and can be applied to metallic solute elements, and Eq. (11) is based on the lever rule for interstitial carbon element. Supplementary Table 3 lists the calculated density contrasts between the mushy zone containing a solid fraction of 0.3 and bulk liquid in various systems.

Supplementary Table 3 shows that the density contrast in the carbon steel is generally an order of magnitude smaller than that in the often used model alloys,

indicating that the thermosolutal convection in the carbon steel is not as strong as that in model alloys. This weak natural convection in widely used steels is, hence, not feasible to drive the occurrence of CS under the same applied solidification conditions as that of model alloys within the classical macrosegregation theory (see the following section of simulations). However, it has been found that, when the content of light solute element is increased up to a certain high level, the density contrast of this kind of steel approaches to those of model alloys. For instance, for high-Si steel with the Si content above 1.0 wt.% (i.e., 27SiMn steel), the density contrast is 2.1, which is very close to the value of -3.7 of the Ga-5 wt.% In alloy (see Supplementary Table 3). Therefore, for this steel, the natural convection may play a key role in the CS formation.

2) Simulations of CS in various systems

In order to further elucidate the effect of density contrast on the CS formation, we have tried to simulate the CS in various systems within the columnar grain solidification model. The solidified grains are supposed to be columnar and the solid movement and shrinkage are not taken into account. The liquid is Newtonian and incompressible while the convection is laminar. The effect of diffusion on the solute transport is also neglected in our current model. The details of the governing equations of the macrosegregation model refers to our previous publication[28], and here we just compile the main equations of conservation of solute, energy, mass and momentum as follows,

Conservation of solute:

$$\frac{\partial[C]}{\partial t}+\nabla\cdot\left(\vec{U}C_l\right)=0 \tag{12}$$

$$[C]=\int_0^{f_s}C_s d\alpha+f_l C_l \tag{13}$$

Conservation of energy:

$$\left[\rho H\right]=f_s\rho_s h_s+(1-f_s)\rho_l h_l \tag{14}$$

$$\frac{\partial[\rho H]}{\partial t}+\nabla\cdot\left(\rho c_p\vec{U}T+\rho\vec{U}\Delta H\right)=\nabla\cdot(\lambda\nabla T) \tag{15}$$

Conservation of mass:

$$\nabla \cdot \left(\vec{U}\right) = 0 \tag{16}$$

Conservation of momentum (x, y directions):

$$\frac{\partial(\rho u)}{\partial t} + \nabla \cdot \left(\rho \vec{U} u\right) = -\frac{\partial P}{\partial x} - \frac{\mu_l}{K} u + \nabla \cdot (\mu_l \nabla u) \tag{17}$$

$$\frac{\partial(\rho v)}{\partial t} + \nabla \cdot \left(\rho \vec{U} v\right) = -\frac{\partial P}{\partial y} - \frac{\mu_l}{K} v + \nabla \cdot (\mu_l \nabla v) + \rho g \left[\beta_T \left(T - T^*\right) + \beta_C \left(C_l - C^*\right)\right] \tag{18}$$

where f_l is liquid fraction, C_s is the solute concentration at the solid side of solid-liquid interface, h_s and h_l are the enthalpies of solid and liquid, respectively; K is the permeability of mushy zone, which is calculated according to the Carman-Kozeny formula when solid fraction f_s is less than the critical solid fraction f_s^c,

$$K = \frac{d_s^2 (1 - f_s)^3}{180 f_s^2} \tag{19}$$

where d_s is the secondary dendritic arm spacing.

In addition, the governing equations are discretized on a staggered Cartesian mesh using a finite volume approach and uniform spaced grids. An explicit scheme of finite volume method is adopted to solve the coupled equations for temperature, concentration, and convection fields. The Solution Algorithm technique is applied to solve the pressure–velocity coupling in the iteration process. Velocity components (u, v) are evaluated on the control volume interfaces. The time step is determined by the maximum velocity at the previous time step. The grid size in all systems should be fine enough to capture all fundamental transport phenomena. Specially, the simulation domains of Sn-Pb, Sn-Bi, Al-Cu, Ga-In, Fe-C and Ni-based superalloy are 100 × 60, 100 × 110, 125 × 100, 50 × 35, 100 × 60 and 50 × 150 mm^2, respectively. The mesh size accordingly are 1 × 1, 1 × 1, 2 × 2, 0.5 × 0.5, 1 × 1 and 1 × 1 mm^2. In addition, the secondary dendrite arm spacings d_s in Sn-Pb, Al-Cu, Sn-Bi, Fe-C, Ga-In and Ni-based superalloy systems are set to be 50, 100, 160, 100, 50, 400 μm, respectively. For Sn-Pb, Sn-Bi, Al-Cu, Ga-In and Fe-C systems, the lateral solidifications are taken into account while the cavity is cooled down from the right-hand side and the remaining sides are thermally insulated. For Ni-based superalloy, the upward

directional solidification has been considered. Initial temperature is the liquidus temperature and the heat transfer coefficients between heat-extraction side and atmosphere are all the same as 300 W $(m^2 \cdot K)^{-1}$. The schematic illustrations of the solidification directions are shown in Supplementary Figure 29.

In our current simulations, we have investigated the impact of solute concentrations on the CS for Al-Cu[4], Ga-In[5], Sn-Bi[10], Sn-Pb[11] and Ni-based superalloy[6]. Among them, two different solute concentrations for each alloy are selected in our simulation procedure: the higher concentration as shown in Supplementary Table 3 refers to the already published literature. But, to simulate the occurrence of CS for the Fe-C binary system we have considered two kinds of solutal expansion coefficients of carbon. One is the normal carbon expansion coefficient which is quite often used in literatures and the other is an artificial value increased by ten times. The former is to mainly check whether or not the CS occurs in normal steels whereas for the latter the simulation is to see how the increased density contrast affects the CS.

For Sn-Pb, Sn-Bi, Al-Cu, Ga-In and Ni-based superalloy we have reproduced well the occurrence of CS (Supplementary Figure 28(a) for Sn-Pb, Supplementary Figure 28(c) for Al-Cu, Supplementary Figure 28(e) for Sn-Bi, Supplementary Figure 28(i) for Ga-In and Supplementary Figure 28(k) for Ni-based superalloy) using the high solute concentrations as what already reported in literatures[4-6,10,11]. However, when low solute concentrations have been considered, the CS disappears (Supplementary Figure 28(b) for Sn-Pb, Supplementary Figure 28(d) for Al-Cu, Supplementary Figure 28(f) for Sn-Bi, Supplementary Figure 28(j) for Ga-In and Supplementary Figure 28(l) for Ni-based superalloy). This fact has demonstrated that the density contrast between solute-enriched melt and bulk liquid determines the occurrence of the CS for the model alloys. The different situation has been observed for the Fe-C system. With normal carbon concentration, our simulations have not uncovered the CS as shown in Supplementary Figure 28(h). When we artificially increase about ten times the carbon solute expansion coefficient, the CS definitely occurs in Supplementary Figure 28(g). These simulations reveal a general conclusion that, on the one hand, in model alloys

of Sn-Pb, Sn-Bi, Al-Cu, Ga-In, and Ni-based superalloy with a normally high solute concentrations, the thermosolutal driven density contrast is large enough, thereby dominating the formation of CS and, on the other hand, in the steels the normal solute concentration is not strong enough to induce the CS. This fact demonstrates that the leading mechanism of the CS in widely used steels is not from the buoyancy force induced by thermosolutal convection. Therefore, the other mechanism via inclusion flotation needs to be illustrated in the steels, as exactly discussed in our main text.

3) Simulations of OIs induced CS formation within multiphase flow approach

In order to further investigate the effect of inclusions on CS formation, the Lagrangian discrete phase model[29] is introduced to the classical macrosegregation model (see the section 2 of Supplementary Note 6) via the Euler-Lagrange approach within the situation that the volume fraction of inclusions is very low in steel melts. The fluid phase is treated as a continuum by solving the time-averaged Navier-Stokes equations (Eq. (16)-(18)), while the dispersed phase is solved by tracking a large number of inclusion particles. The fluid phase influences the particulate phase via dragging and convection, while the particulate phase disturbs the fluid phase via the source terms of momentum. Therefore, the force induced by the flotation of solid particles is included in the source term of the momentum equations, such as in the gravity direction and the Eq. (18) can be rewritten as,

$$\begin{aligned}\frac{\partial(\rho v)}{\partial t}+\nabla\cdot\left(\rho\vec{U}v\right)=-\frac{\partial P}{\partial y}+\nabla\cdot(\mu_l\nabla v)\\+\rho g\left[\beta_T\left(T-T^*\right)+\beta_C\left(C_l-C^*\right)\right]+\frac{K_{pl}}{f_l}(v_p-v)\end{aligned}\qquad(20)$$

where v_p is the velocity of a particle; K_{pl} is the interfacial friction coefficient, which is modified from the following Gidaspow correction[30],

$$K_{pl}=\frac{3}{4}C_D\frac{f_p f_l\rho}{d_p}\left|v_p-v_l\right|f_l^{-2.65}+150\frac{f_s^2\mu_l}{f_l d_s^2}\qquad(21)$$

here, the first and second terms represent the contributions of inclusion particles and dendritic network, respectively. C_D is the drag coefficient and is chosen equal to $24/Re_D$ (Re_D is the relative Reynolds number) in the current paper. f_p is the occupied

area fraction of particle in the local cell and d_p is the diameter of a given particle.

The moving velocity of a solid particle in melt depends on the buoyancy force, gravity force and the drag force. Hence, the trajectory of a discrete phase particle is predicted by integrating the forces balance on the particle. This can be expressed within a Lagrangian reference framework,

$$\frac{d\mathbf{u}_{\mathrm{P}}}{dt} = f_D\left(\mathbf{u}_{\mathrm{P}} - \mathbf{u}\right) + \frac{\mathbf{g}\left(\rho_p - \rho_l\right)}{\rho_p} \tag{22}$$

where $\mathbf{u_p}$ and $\mathbf{u}$ are the velocity vectors of solid particle and liquid, respectively, and $\mathbf{g}$ is the acceleration of gravity, ρ_p and ρ_l are the density of particle and liquid, respectively. ρ_p is equal to 3640 kg m^{-3} for alumina. It is assumed that the particle is a solid sphere, hence f_D can be given by

$$f_D = \frac{18\mu_l}{\rho_p d_p^2}\frac{C_D Re_D}{24} \tag{23}$$

It is worth noting that in current simulations an alumina particle will be trapped once it arrives to the cavity boundaries or the local solid fraction exceeds 0.5. Spherical particles are released randomly in the cavity at the beginning of solidification. The phenomena of agglomeration and growth of particles are not taken into account. The resolution of velocity-pressure coupling in the multiphase flow system is performed by SIMPLE algorithm. The grid size is 1×1 mm^2. All the solidification conditions and physical parameters are the same as the simulation of Fe-0.36 wt.% C steel in the section 2 of Supplementary Note 6.

Numerical simulations have been performed to investigate the influence of OIs on CS formation with different sizes and initial numbers of particles. Supplementary Figure 24 shows the final macrosegregation patterns in the cavity with different sizes of particles between 2 and 30 μm. Before solidification, 500 particles are injected into the melt randomly in space. From Supplementary Figure 24a to Figure 24f by increasing the particle diameter, the CS becomes more apparent and severe. The main feature is the increased CS strips and the serious segregation of solute concentration. Importantly, the calculations also found that, when the particle size is too large the CS disappears again, as illustrated in Supplementary Figure 24f. Hence, in terms of our

simulations it has been found that the CS occurrence would require the proper range of the particles' dimensions. We carefully check this range, indicating the particles with the diameters from 5 μm to 30 μm are the best choice to induce the CS formation. In addition, we also analyzed the effect of the quantity of the particles on the CS formation, as shown in Supplementary Figure 25. Here, all the particles have the same diameter of 15 μm. It can be clearly seen that with increasing the number of the initial particles the CS becomes more obvious as demonstrated in Supplementary Figure 25a to Supplementary Figure 25c.

SUPPLEMENTARY METHODS: Experimental ingots

In the main text, we have already listed the details of the eight ingots from **I** to **VIII**. In order to provide the integrated evidence, here we have further extended the series of the experimentally sectioned ingots weighted in the range among 0.5, 5.0, 5.8, 14, 16, 20, 69, 100, 234, 535 and 650 tons, as included in Supplementary Table 1. Our extended experiments and analysis further reveal the consistent and firm proofs that the occurrence of CS is mainly determined by the total oxygen concentration (T.O) in steel ingots and the deoxidation processes. If T.O is controlled to a low level (i.e., 1.0×10^{-3} wt.%), the formation of CS is significantly reduced, or completely eliminated even via the Al-deoxidation (AD) technique (see **IX-XIV** in Supplementary Table 1). In addition, as illustrated in Supplementary Table 1 for the ingots of **XV**, **XVI**, **XVII** and **XVIII** even when the T.O is as high as $(1.2\sim1.5) \times 10^{-3}$ wt.% the CS can be completely eliminated through the vacuum carbon deoxidation technique (VCD). In these Supplementary experimental ingots the CS can not be observed only if the oxide-based inclusion is prohibited due to a low oxygen concentration (typically, around 1.0×10^{-3} wt.%) through vacuum carbon deoxidation (VCD) technique. In contrast, our experimental analysis has also revealed that, if using the AD technique in the final stage of the refining process, the CS will perhaps appear because the oxide-based inclusions can be easily formed via the AD technique. These experiments and analysis clearly demonstrate that the traditionally recognized thermosolutal convection does not play a crucial role in forming the CS in steel ingots. Hence, it would be highly difficult to eliminate the CS, by just reducing the density contrasts between the melt enriched with C, Si, Mn, S and P solutes and bulk melt if the oxide-based inclusions exist. In contrast, as illustrated in our main text the consistent scientific fact is that, if during the upstream metallurgical process the oxygen concentration is controlled to a highly low level (typically, 1.0×10^{-3} wt.%), the CS can be drastically reduced, even completely disappeared, mainly because the oxide-based inclusions are prohibited due to the low oxygen concentration. This phenomenon has been long-term neglected in the past fifty years. Here, our work is providing the clear evidence that the crucial origin of the occurrence of CS in steels is

the existence of oxide-based inclusions. The details of the experimental ingots from **IX** to **XVIII** have been provided in Supplementary Table 1.

SUPPLEMENTARY References

1. Wang, J. Q., Fu, P. X., Liu, H. W., Li, D. Z. & Li, Y. Y. Shrinkage porosity criteria and optimized design of a 100-ton 30Cr2Ni4MoV forging ingot. *Mater. Des.* **35**, 446-456 (2012).
2. Aldebert, P. & Traverse, J. P. Neutron diffraction study of structural characteristics and Ionic mobility of α-Al_2O_3 at high temperatures. *J. Am. Ceram. Soc.* **65**, 460 (1982).
3. Kajikawa, K., Suzuki, S., Takahashi, F., Yamamoto, S., Suzuki, T., Ueda, S., Shibata, T. & Yoshida, H. Development of 650-ton-class ingot for turbine rotor shaft forging application. In *1st International Conference on Ingot Casting, Rolling and Forging* (Aachen, Germany, 2012).
4. Mehrabian, R., Keane, M. A. & Flemings, M. C. Experiments on macrosegregation and freckle formation. *Metall. Trans.* **1**, 3238-3241 (1970).
5. Koster, J. N. & Derebail, R. A threshold for onset of natural convection in binary metallic alloys. Int. *J. Heat Mass Transfer* **32**, 489-498 (1997).
6. Schneider, M. C., Gu, J. P., Beckerman, C., Boettinger, W. J. & Kattner, U. R. Modeling of micro- and macrosegregation and freckle formation in single-crystal nickel-base superalloy directional solidification. *Metall. Mater. Trans. A* **28**, 1517-1531 (1997).
7. Guillemot, G., Gandin, C. A. & Bellet, M. Interaction between single grain solidification and macrosegregation: Application of a cellular automaton—Finite element model. *J. Cryst. Growth* **303**, 58-68 (2007).
8. Založnik, M., Kumar, A. & Combeau, H. An operator splitting scheme for coupling macroscopic transport and grain growth in a two-phase multiscale solidification model: Part II – Application of the model. *Comput. Mater. Sci*. **48**, 11-21 (2010).
9. Kumar, A., Walker, M. J., Sundarraj, S. & Dutta, P. Metall. Grain floatation during equiaxed solidification of an Al-Cu alloy in a side-cooled cavity: Part II—Numerical studies. *Mater. Trans. B* **42,** 783-799 (2011).
10. Sawada, T., Oikawa, K. & Anzai, K. Three-dimensional numerical simulation of channel segregation in directionally solidified Sn-20 mass% Bi ingot. *Tetsu-to-Hagane* **99**, 135-140 (2013).
11. Hebditch, D. J. & Hunt, J. D. Observations of ingot macrosegregation on model. *Metall. Trans.* **5**, 1557-1564 (1974).

12. Flemings, M. C. Solidification Processing (McGraw-Hill, New York, 1974).

13. Procast User Manual Version 2009. 1. ESI group. The virtual try-out space company, (2009).

14. Jin, Y., An, H. P., Ma, P. & Sun, H. Y. Preliminary study of solidification property in large steel ingot. *Heavy Casting and Forging* **1**, 5-8 (2011) (In Chinese).

15. Hamilton, R. W., Forster, M. F., Dashwood, R. J. & Lee, P. D. Application of X-ray tomography to quantify the distribution of TiB_2 particulate in aluminium. *Scr. Mater.* **46**, 25-29 (2002).

16. Kurita, T., Uchida, K. & Oshiyama, A. Atomic and electronic structures of α-Al_2O_3 surfaces. *Phys. Rev. B* **82**, 155319 (2010).

17. Wallin, E., Andersson, J. M., Münger, E. P., Chirita, V. & Helmersson, U. Ab initio studies of Al, O, and O_2 adsorption on α-Al_2O_3(0001) surfaces. *Phys. Rev. B* **74**, 125409 (2006).

18. Tang, W., Sanville, E. & Henkelman, G. A grid-based Bader analysis algorithm without lattice bias. *J. Phys. Condens. Matter.* **21**, 084204–084207 (2009).

19. Sanville, E., Kenny, S. D., Smith, R. & Henkelman, G. Improved grid-based algorithm for Bader charge allocation. *J. Comput. Chem.* **28**, 899-908 (2007).

20. Karma, A. Phase-field formulation for quantitative modeling of alloy solidification. *Phys. Rev. Lett.* **87**, 115701 (2001).

21. Echebarria, B., Folch, R., Karma, A. & Plapp, M. Quantitative phase-field model of alloy solidification. *Phys. Rev. E* **70**, 061604 (2004).

22. Worster, M. G. Convection in mushy layers. *Annu. Rev. Fluid Mech.* **29**, 91–122 (1997).

23. Li, R. On multi-mesh h-adaptive methods. *J. Sci. Comput.* **24**, 321-341 (2005).

24. Chen, Y., Nguyen-Thi, H., Li, D. Z., Bogno, A.-A., Billia, B. & Xiao, N. M. Influence of natural convection on microstructure evolution during the initial solidification transient: comparison of phase-field modeling with in situ synchrotron X-ray monitoring data. *IOP Conf. Ser.: Mater. Sci. Eng*. **33**, 012102 (2012).

25. Guermond, J.-L. & Quartapelle, L. On stability and convergence of projection methods based on pressure Poisson equation. *Int. J. Numer. Meth. Fluids* **26**, 1039-1053(1998).

26. Campbell, J., *Castings*, 2nd ed. (Butterworth-Heinemann, Burlington, 2003).

27. Yuan, L. & Lee, P. D. A new mechanism for freckle initiation based on microstructural level simulation. *Acta Mater.* **60**, 4917–4926 (2012).

28. Liu, D., Sang, B., Kang, X. & Li, D. Z. Effect of alloy insert on channel segregation during solidification of Sn-10 wt pct Bi alloy. *Metall. Mater. Trans. B* **42**, 210-223 (2010).

29. Gouesbet, G. & Berlemont, A. Eulerian and Lagrangian approaches for predicting the behaviour of discrete particles in turbulent flows. *Prog. Energy Combust. Sci.* **25**, 133-159 (1998).

30. Gidaspow, D. Multiphase flow and fluidization: continuum and kinetic theory description (Academic Press, New York, 1994).
